# Shape, membrane morphology, and morphodynamic response of metabolically active human mitochondria revealed by scanning ion conductance microscopy

**DOI:** 10.3762/bjnano.16.73

**Published:** 2025-06-30

**Authors:** Eric Lieberwirth, Anja Schaeper, Regina Lange, Ingo Barke, Simone Baltrusch, Sylvia Speller

**Affiliations:** 1 Institute of Physics, University of Rostock, 18059 Rostock, Germany; 2 Department of Life, Light and Matter, University of Rostock, 18059 Rostock, Germany; 3 Institute of Medical Biochemistry and Molecular Biology, Rostock University Medical Center, 18057 Rostock, Germany

**Keywords:** HeLa, metabolically active, mitochondria, morphodynamics, scanning ion conductance microscopy

## Abstract

Mitochondrial network dynamics play a key role in enabling cells to adapt to environmental changes. Fusion and fission of mitochondria, as well as their contact with other organelles, are central processes. Consequently, the outer membrane, which separates the mitochondrion from the cytoplasm, has become a focus of investigation. We analysed metabolically active mitochondria from HeLa cells using scanning ion conductance microscopy to generate nanoscopically resolved, three-dimensional topographies. Our measurements reveal the diversity of mitochondrial shapes. Moreover, a morphodynamic effect was identified, the magnitude of which depends on mitochondrial viability. This method, applied for the first time to mitochondria, shows potential for visualising the morphodynamic responses of mitochondria to their local environment. The similarities between the nanopipette in the measurement setup and the microtubules in the cellular context are discussed as the basis for the hypothesis.

## Introduction

Mitochondria are essential organelles in eukaryotic cells, primarily responsible for energy supply. They have an endosymbiotic origin, resulting from the incorporation of an ancestral prokaryote into another prokaryotic host approximately 1.8 billion years ago [1–3]. This symbiotic relationship gave rise to mitochondria, which possess a double membrane, dividing the organelle into four compartments, namely, the outer membrane, the intermembrane space, the inner membrane (which is invaginated and forms the so-called cristae), and the matrix. In addition to hosting catabolic metabolic pathways and the citric acid cycle, mitochondria facilitate oxidative phosphorylation, which generates the energy carrier adenosine triphosphate (ATP). Indicators of mitochondrial metabolic activity include a high membrane potential and oxygen consumption [4–5].

Recent research emphasises that mitochondria should not be studied as isolated compartments but as components of a highly dynamic network. This mitochondrial network undergoes constant restructuring through processes of fusion and fission and interacts extensively with other organelles. Mitochondria are moved along the cytoskeleton and undergo fusion and fission [6–9]. These processes are integral to maintaining cellular homeostasis [10], and disruptions in mitochondrial function are implicated in numerous diseases, including Parkinson’s disease [11–13], cardiovascular diseases [14–16], Alzheimer’s disease [12,17–18], obesity [19], and diabetes mellitus [20–21]. While the inner mitochondrial membrane and its cristae structure have been extensively studied, comparatively little is known about the outer mitochondrial membrane. The outer membrane, in direct contact with the cytosol and the other organelles, plays a crucial role in cellular processes and warrants further investigation.

Scanning probe microscopy (SPM) methods, such as atomic force microscopy (AFM), have been employed to image mitochondria in liquid, showing features of both the inner and outer membrane [22–24]. However, AFM measurements are influenced by the cantilever–sample interaction, often leading to an underestimation of mitochondrial apparent height due to applied cantilever pressure [22,25]. Similarly, scanning electron microscopy (SEM) offers high-resolution imaging but requires mitochondria to be chemically fixed, stained, and sectioned, which precludes the study of metabolically active organelles [26].

Scanning ion conductance microscopy (SICM) enables three-dimensional visualisation of living cells in their native state [27]. Pioneered by Hansma et al. [28], the method has been continuously refined since the 1990s [27,29–30]. SICM utilises a nanopipette with a sub-micrometer aperture, which is brought near the sample surface via piezo actuators in an electrolyte bath. A voltage applied between two Ag/AgCl electrodes, one in the pipette and the other in the bath, generates an ion current. If the pipette approaches the sample, the ionic current drops sharply due to reduction of ion cross section. This decrease triggers the feedback system to stop the approach at a predefined setpoint current, effectively determining the *z*-position of the sample at that location. By scanning the sample pixel-by-pixel in hopping mode, a three-dimensional topography is acquired. SICM is particularly well suited for biological applications, as it is a non-contact method with minimal interaction forces, preserving the native state of the sample [27]. To date, SICM investigations of mitochondria have been limited to subsarcolemmal mitochondria, specifically in cardiomyocytes. These studies include analyses of mitochondrial compliance and the role of mitochondria in heart failure, achieved through a combination of SICM and confocal microscopy. An overpressure was applied to the pipette, enabling observations of mitochondria through the cell membrane [31–32]. Another study demonstrated the use of a nanopipette for extraction of small mitochondrial subpopulations from fibroblasts for next-generation genome sequencing [33]. However, metabolically active, isolated mitochondria and their surface morphology at nanometer resolution have not been reported using SICM.

In this work, we employ SICM to investigate the shape, dynamics, and nanomorphology of metabolically active, isolated mitochondria, with a specific focus on the outer mitochondrial membrane. Furthermore, we identify and characterise two distinct dynamic effects observed in time-dependent topography data. One of these effects appears to be associated to the dynamics of the mitochondria’s morphology (in the following referred to as morphodynamics, cf. [6]) and their contact with the measurement environment. Finally, we propose hypotheses to explain the observed effects.

## Results

**Figure 1:**
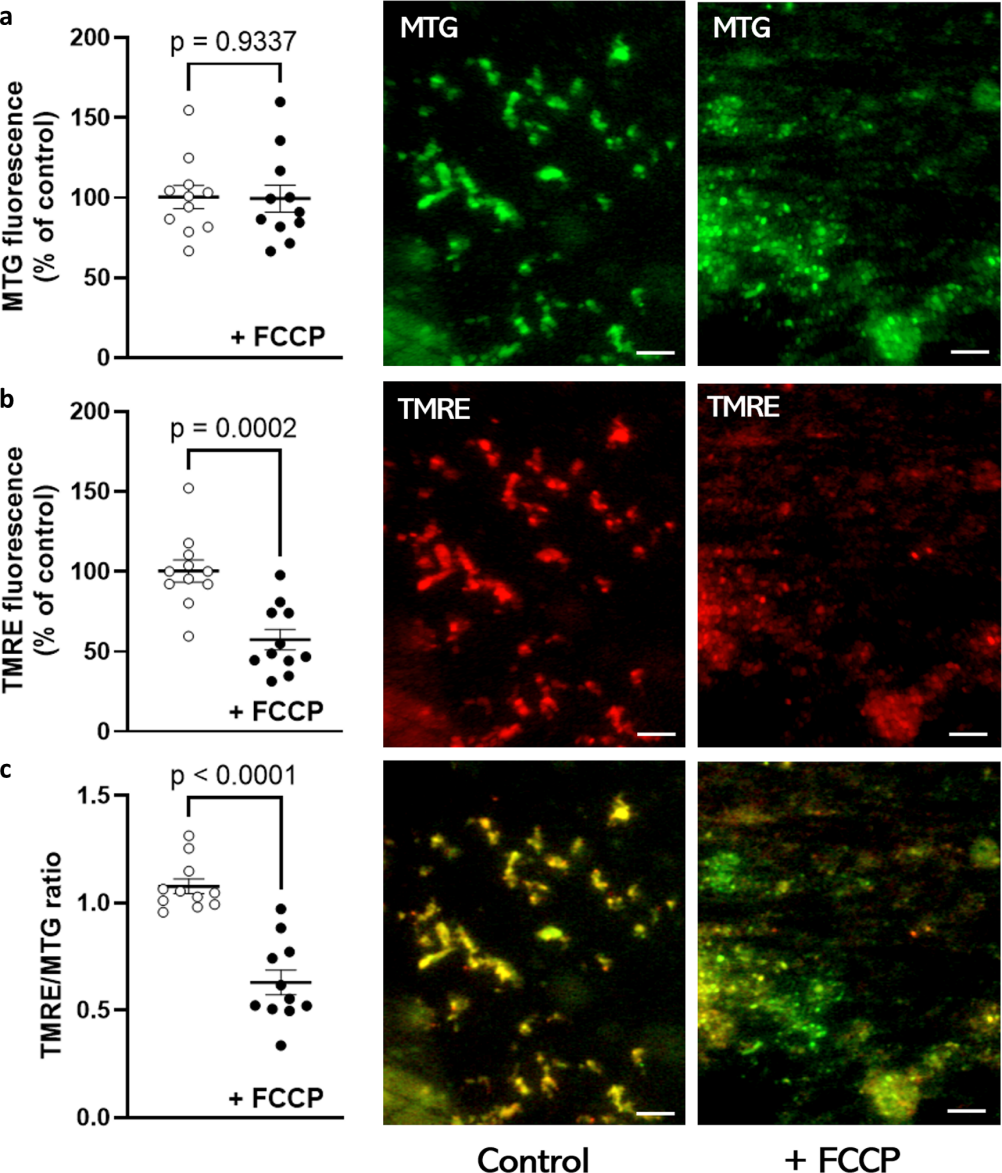
Characterisation of isolated mitochondria by fluorescence microscopy. Isolated mitochondria were stained with MTG (a) and TMRE (b) and investigated for 30 min with and without treatment with 5 μM FCCP. The fluorescence intensity of both dyes was quantified in ten representative individual mitochondria. Both, TMRE fluorescence and the TMRE/MTG ratio (c) significantly decreased after FCCP treatment compared to the control (Student’s *t*-test). Scale bars: 2 μm.

Viability of the investigated mitochondria was analysed by microscopy and oxygen consumption measurement. Figure 1 shows isolated mitochondria by nitrogen cavitation [34] and subsequently labelled by MitoTracker™ Green FM (MTG, Figure 1a) and tetramethylrhodamine ethyl ester perchlorate (TMRE, Figure 1b). TMRE accumulates only in the metabolically active mitochondria with high membrane potential (Figure 1b, control in centre column). After treatment with FCCP, depolarisation occurs (Figure 1b, FCCP in the right column) and TMRE fluorescence decreases. MTG labels mitochondria independently from the membrane potential. Therefore, the TMRE/MTG ratio decreases significantly after FCCP treatment (Figure 1c).

In addition, oxygen consumption was measured. An oxygen consumption rate of 211.0 ± 10.16 μmol·L^−1^ was recorded within 10 min in Krebs–Ringer medium with CaCl_2_, 5.5 mmol·L^−1^ glucose and 0.1% BSA (mass fraction), regardless of the isolation method. After storing the mitochondrial fractions at −80 °C for four days to three months, oxygen consumption was only slightly reduced to 182.8 ± 16.88 μmol·L^−1^ per 10 min. Thus, freezing and thawing have little effect on mitochondrial performance. Because the additives are not suitable for measurement in SICM, oxygen consumption measurements are also performed without. Under this condition, oxygen consumption of mitochondria is reduced to 123.8 ± 9.76 μmol·L^−1^ per 10 min, but the organelles were proven viable. In addition to functional analysis, the mitochondrial fractions obtained were analysed using Western blot.

**Figure 2:**
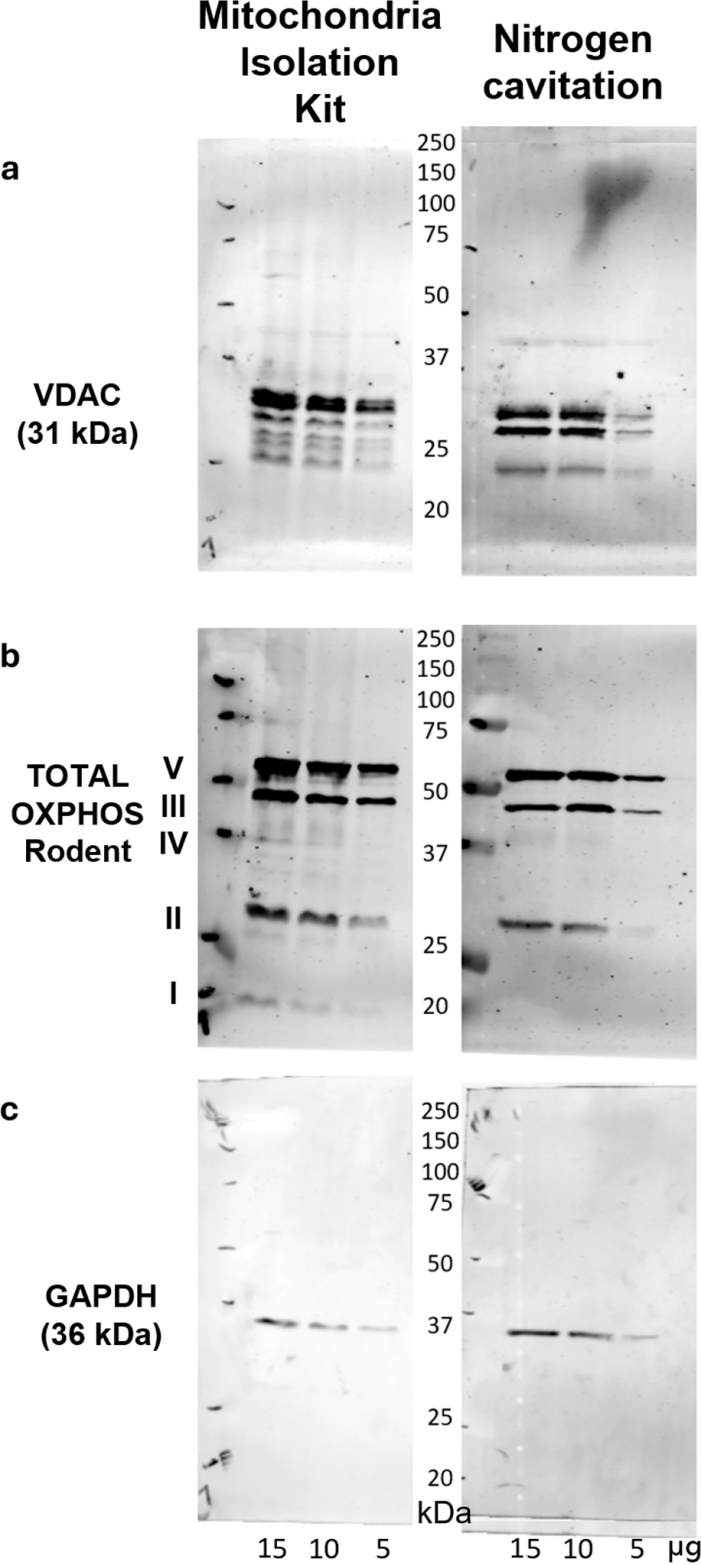
Characterisation of isolated mitochondria by Western blot analyses. Detection of specific proteins of (a) the outer (voltage-dependent anion channel, VDAC) and (b) the inner (complexes of the respiratory chain and ATP synthesis, OXPHOS) mitochondrial membrane demonstrate the quality of mitochondria of both isolation methods. (c) A minor signal for glyceraldehyde-3-phosphate dehydrogenase (GAPDH) shows low contamination by cytosolic proteins.

The voltage-dependent anion channel of the outer mitochondrial membrane (Figure 2a) as well as complexes of the respiratory chain and ATP synthesis (Figure 2b), which are localised in the inner membrane, could be detected in significantly higher quantities than the cytosolic abundant enzyme glyceraldehyde-3-phosphate dehydrogenase (Figure 2c).

We analysed 21 metabolically active mitochondria from HeLa cells with SICM, resulting in a data set of 65 measurements (some mitochondria were measured several times). Shape analysis is based on the roundness of the projected area of the mitochondrion in the *xy*-plane. Classification is performed by comparing the mitochondrial perimeter *p*_m_ with the perimeter *p* of a circle having the same area as the mitochondrial projection. This yields a parameter *R*, which quantifies the deviation from a perfect circle:


[1] $$R=\frac{p_{\text{m}}}{p}.$$

**Figure 3:**
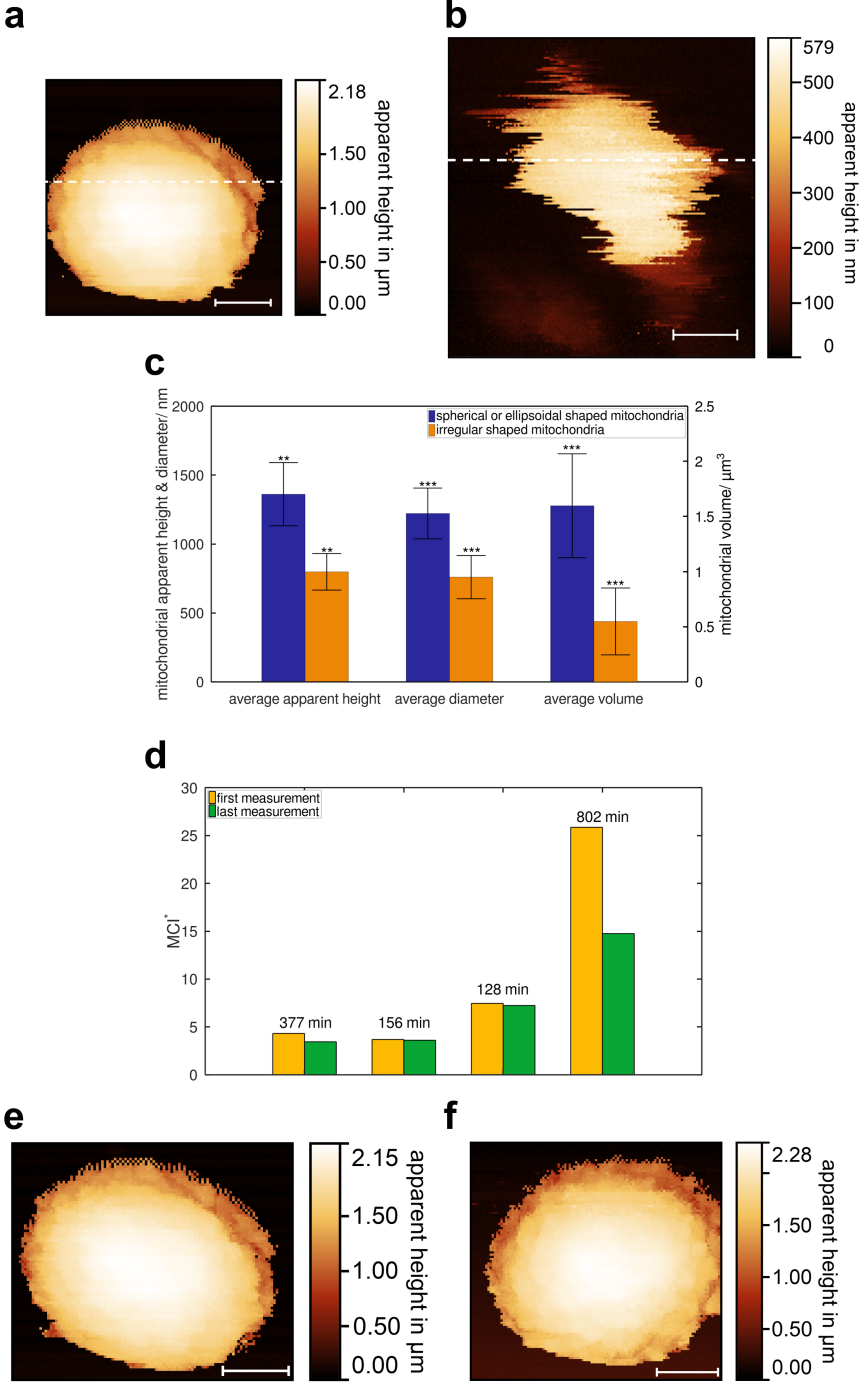
False-colour topographies and statistical analyses to illustrate the geometric properties of metabolically active mitochondria, isolated via nitrogen cavitation, dashed lines show location of line profiles, line profiles display in Figure 7a,b. (a) Spherical mitochondrion with a diameter of approx. 2 μm, 128 × 128 pixels, measurement time *t* = 30 min. (b) Irregularly shaped mitochondrion with a diameter of approx. 0.3 μm, 256 × 256 pixels, *t* = 45 min. (c) Statistical analyses for spherical to ellipsoidal shaped mitochondria (blue) and irregularly shaped mitochondria (orange), **: *p <* 0.05, ***: *p <* 0.1, Welch’s *t*-test. (d) Bar graph showing MCI^*^, first (yellow) and last (green) measurement of four repeatedly measured mitochondria, time elapsed over bars, MCI^*^ decreases in all cases, results suggest a trend towards a more spherical shape, reduced statistical significance. (e) First measurement of a spherical mitochondrion, 128 × 128 pixels, *t* = 30 min. (f) Seventh measurement of the same spherical mitochondrion as in (e), 128 × 128 pixels, *t* = 30 min, elapsed time between both measurements is 6 h and 17 min. Scale bars: (a, e, f) 500 nm; (b) 100 nm.

An *R*-value of 1 indicates a circular projection. Shapes with a deviation of max. 15% are classified as ’spherical or ellipsoidal’, while all others are considered ’irregular’. The shape of eleven metabolically active mitochondria is close to spherical or ellipsoidal (Figure 3a). We also observe ten cases of more irregular shapes (Figure 3b). Additional examples illustrating the diversity of mitochondrial shapes can be found in Section S1 of Supporting Information File 1. The measured diameters (for irregular shapes: the maximum expansion in the slow scan direction) ranged from 0.2 to 2.6 μm, with an average of 1.2 μm. Apparent heights varied between 0.2 and 2.8 μm, with an average of 1.2 μm. The topography shows shallow irregular undulations with an amplitude and spatial dimension of approximately 100 nm.

Statistical analyses comparing the spherical or ellipsoidal with the irregular mitochondria are provided in Figure 3c. The average values of apparent height, diameter, and volume are compared. Irregular mitochondria tend to be smaller with reduced statistical significance (Welch’s *t*-test).

The size distribution of metabolically active mitochondria is bimodal, that is, diameters predominantly fell into two categories, either below 1.0 μm or above 1.5 μm, with few measurements in the intermediate range (see Supporting Information File 1, Section S2 for a histogram of the measured diameters). To investigate potential shape changes because of the measurement, four mitochondria were measured repeatedly over time. Shape changes were quantified using a metric according to mitochondrial complexity index (MCI) [9], as it is suitable for assessing both regular and irregular morphologies. It is modified to


[2] $${\text{MCI}}^{*}=\frac{{\left(\text{SA}\right)}^{\frac{3}{2}}}{6\sqrt{\pi }V},$$

where SA is the surface area (computed by triangulation) and *V* is the volume (integral of the apparent height over *x*- and *y*-dimensions). The pre-factor in the denominator is changed. Now, MCI^*^ = 1 is valid for a sphere. Under the given experimental conditions, it is not possible to measure an entire sphere, as the probe is always approaching from the top. The closest approximation is a hemisphere, corresponding to 
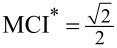
. Since the bottom surface is inaccessible, only the evolution of the MCI^*^ over time is considered. If the MCI^*^ approaches 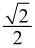 as time progresses, the mitochondrion tends toward a more spherical shape.

Figure 3d presents a bar graph of the MCI^*^ for the four data sets. The time elapsed between the completion of the scans is shown above the bars. In all cases, MCI^*^ decreased, indicating that mitochondria tend to adopt a more spherical shape during repeated measurements. Statistical analysis using a one-sided *t*-test at a 90% confidence level provides significant evidence for this decrease, as the null hypothesis is rejected. However, when applying the commonly used confidence level of 95%, the null hypothesis cannot be rejected. The results suggest a trend towards a more spherical shape, albeit with reduced statistical significance. Diameter and apparent height remain consistent over time. One exemplary data set is depicted in Figure 3e,f. Figure 3e shows the false-colour topography of a mitochondrion with a initial diameter of 1.96 μm and a maximum apparent height of 2.15 μm. After an elapsed time of 6 hours and 17 minutes, the same mitochondrion (Figure 3f) exhibits a slightly increased diameter of 2.05 μm and a maximum apparent height of 2.28 μm.

**Figure 4:**
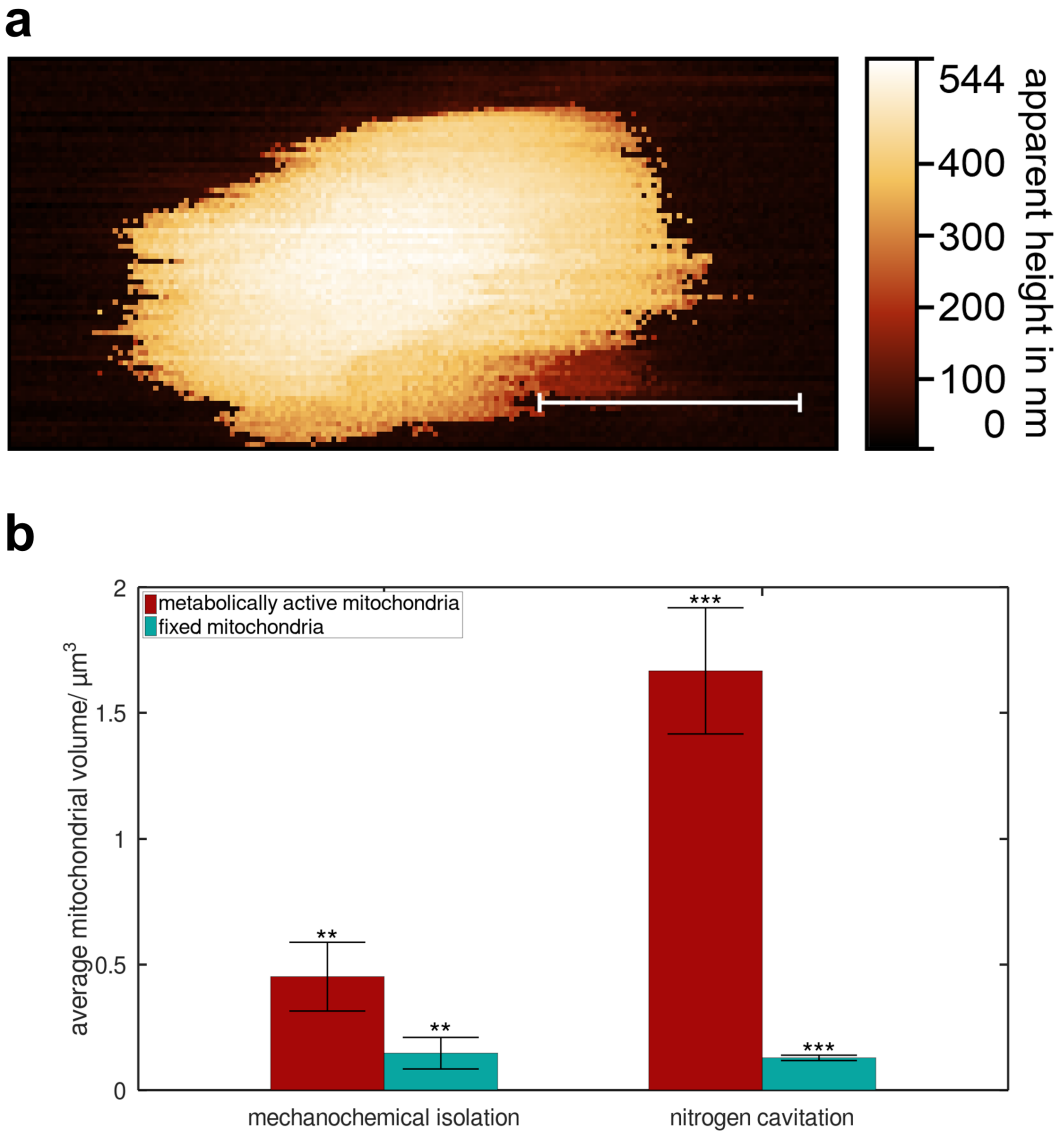
False-colour topography of a fixed mitochondrion and statistical analysis to illustrate the geometric properties of metabolically active vs fixed mitochondria for different isolation methods. (a) Fixed mitochondrion, 256 × 256 pixels, measurement time *t* = 60 min, isolated via mechanochemical protocol, scale bar: 200 nm. (b) Statistical analyses for average mitochondrial volume of different isolation methods, red: metabolically active mitochondria, turquoise: fixed mitochondria, **: *p >* 0.05, ***: *p <* 0.001, Welch’s *t*-test.

To explore the relationship between shape and metabolic activity, we also measured seven fixed mitochondria using SICM (Figure 4a). These fixed mitochondria had diameters ranging from 0.2 to 1.4 μm (average: 0.8 μm) and apparent heights between 0.2 and 1.4 μm (average: 0.7 μm), indicating that they were generally smaller. A statistical analysis is provided in Figure 4b. The mitochondrial volume was used as a shape-independent parameter and was calculated from the obtained topography data without relying on any specific model. Mitochondria isolated via mechanochemical pathway (left bars) show no significant difference in average mitochondrial volume between metabolically active and fixed mitochondria at a 95% confidence level. In contrast, mitochondria isolated through nitrogen cavitation (right bars) demonstrated a statistically significant difference in average mitochondrial volume (*p <* 0.001, Welch’s *t*-test).

### Edge memory effect

Metabolically active mitochondria possess the ability to respond to external stimuli. In cells, these responses are reflected in alterations to the shape, size, and number of the mitochondria, which in turn influence their metabolic performance. These fast adaptive behaviours are referred to as morphodynamic effects, or morphodynamics for short [6]. In the context of SICM, morphodynamic changes in mitochondrial shape can be directly observed. Because of the comparatively long measurement time in relation to other SPM techniques, the time constant is sufficiently large to allow a morphodynamic response. Morphodynamic effects are particularly pronounced at the edges of mitochondria. When a mitochondrion adopts a more spherical shape through morphodynamic activity, the maximum apparent height increases slightly, while its extension in the *xy*-plane decreases. To analyse the possible three-dimensional morphodynamic responses of mitochondria, we derived a parameter TEV from the SICM images:


[3] $$\text{TEV}=\Delta x\cdot \Delta y\cdot \sum_{i}\left|z_{i}^{\text{bwd}}-z_{i}^{\text{fwd}}\right|,$$

where Δ*x* and Δ*y* are the edge lengths of the pixel (which are constant within each scan), and 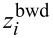 and 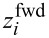 the apparent heights at pixel *i* of the backward and forward scan, respectively. The modulus ensures that positive and negative values of the difference 
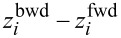
 contributes to TEV.

**Figure 5:**
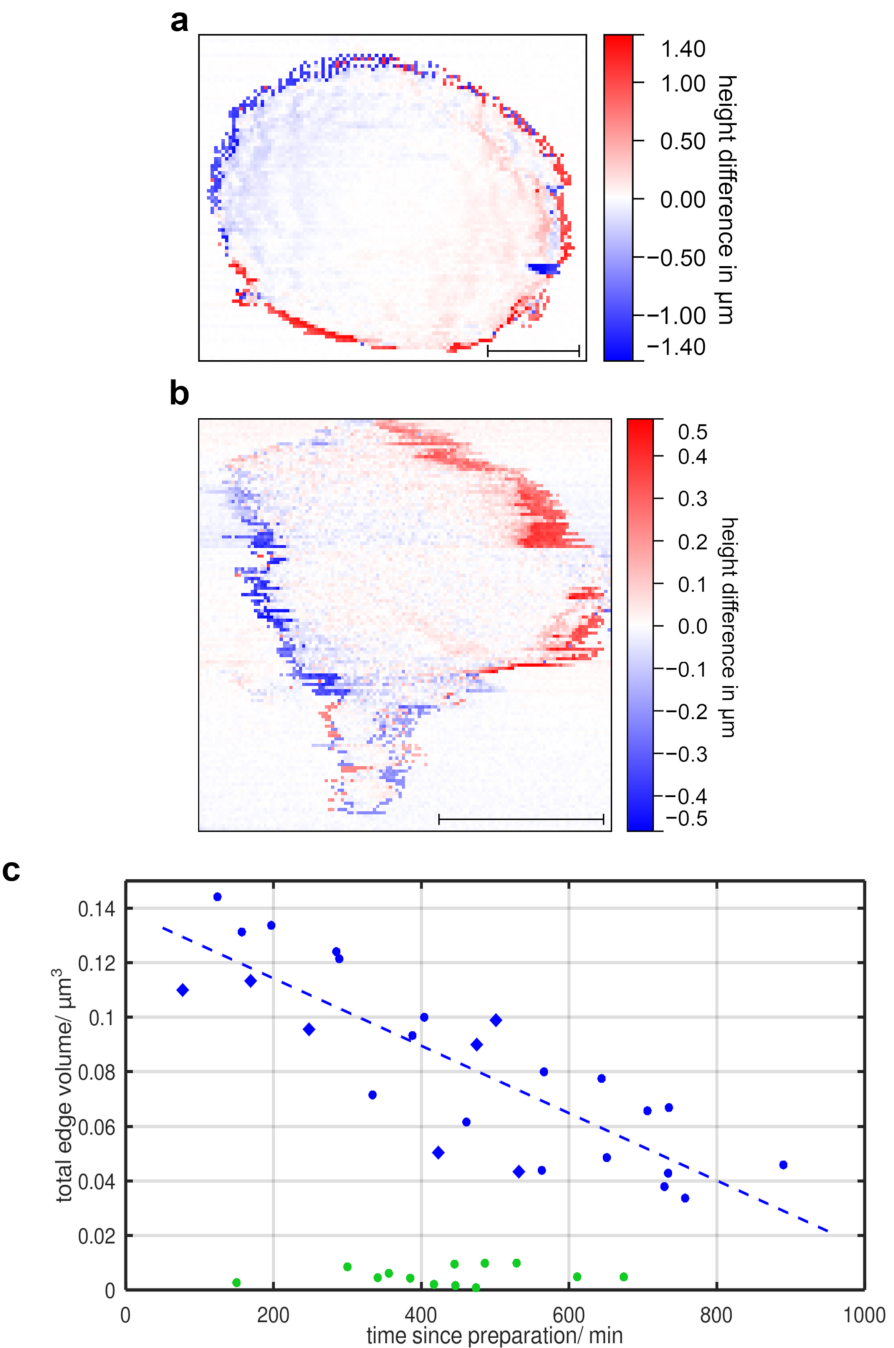
Measurements illustrating the edge memory effect. (a) Difference image of a larger mitochondrion, 128 × 128 pixels, *t* = 30 min. (b) Difference image of a smaller mitochondrion, 128 × 128 pixels, *t* = 30 min. (c) Graph showing the TEV depending on the time passed since preparation. The values can be grouped. The groups are labelled with different colours (blue and green). Data points labelled as a diamond are measurements, which show more than 90% but less than 100% of the mitochondrion due to drift and self-motion (TEV nominally greater than measured). Examples are shown in Supporting Information File 1, Section S3, Figures S7 and S8. Scale bars: (a) 500 nm; (b) 200 nm.

At first glance, the TEV may appear to be an arbitrary parameter. However, as demonstrated below, it is a suitable metric for describing morphodynamics. Fundamentally, the TEV quantifies morphological changes along the pipette’s scanning trajectory. Whenever the apparent heights (*z*-values) at a given point (*x*, *y*) differ between the associated forward and backward scans, this discrepancy contributes to the TEV. For compact objects, such differences primarily arise when the lateral position of the object’s edges changes, as illustrated in Figure 5a,b. Additional so-called difference images of various metabolically active mitochondria, as well as a time series measurement of a single mitochondrion, are provided in Supporting Information File 1, Section S3. Accordingly, this parameter is referred to as the total edge volume (TEV).

In the field of SPM, several mechanisms are known to influence parameters like the TEV without necessarily being related to the morphodynamics of the mitochondrion. In general, contributions to TEV(*t*_P_) in SPM experiments can include artefacts such as (mechanical) hysteresis effects caused by the elastic behaviour of the measuring tip or the mechanical components of the SPM setup, as well as repeated displacements of the object during the scan of each line. Drift phenomena are considerable, too, which are primarily caused by the lateral measurement process and result in considerable lateral forces. However, in SICM operating in hopping mode, the nanopipette approaches the sample vertically at each pixel, substantially reducing lateral forces acting on the sample [27,35]. Consequently, the TEV is a useful metric for characterising the morphodynamic response of mitochondria.

Figure 5c illustrates the correlation between the measured TEV and the time elapsed since sample preparation *t*_P_. The time elapsed since sample preparation begins after the thawing of the isolated mitochondria. The observed time dependence of TEV(*t*_P_) (blue markers) follows a linearly decreasing trend, represented by the blue dotted line. This trend supports the suitability of the TEV as a parameter for characterising morphodynamics. The hysteresis effects mentioned earlier are always time-independent.

**Figure 6:**
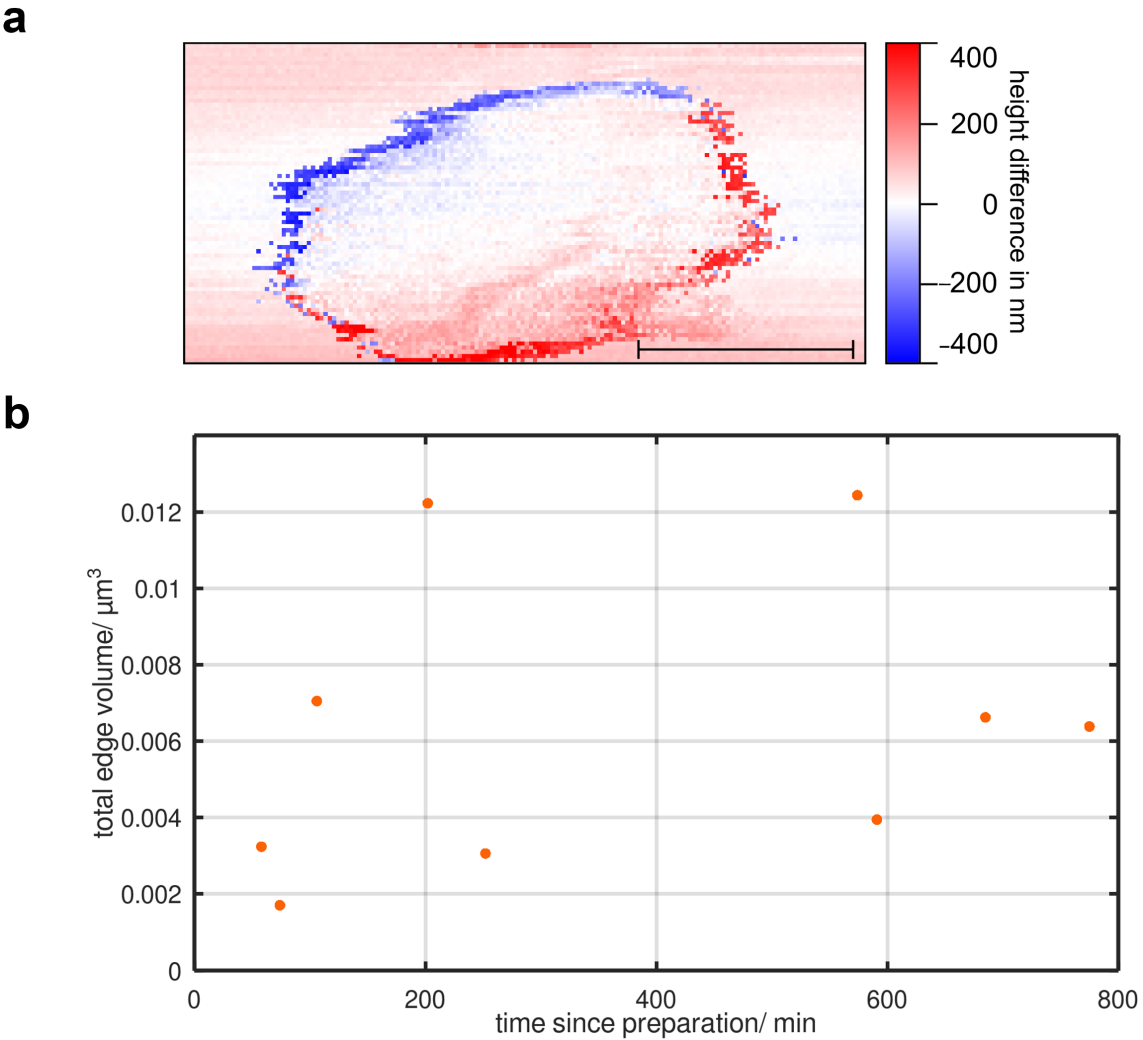
Measurements illustrating drift phenomena in SPM. (a) Difference image of a fixed mitochondrion, 256 × 256 pixels, *t* = 50 min, scale bar: 200 nm. (b) Graph showing the TEV of fixed mitochondria depending on the time passed since preparation.

The TEV is still not zero for non-viable objects like fixed mitochondria, as shown in Figure 6a,b. The TEV for metabolically active mitochondria is approximately ten times higher, indicating that the mentioned artefacts are negligible in this context. Therefore, we interpret the TEV as an indicator of mitochondrial morphodynamics and metabolic activity, a phenomenon we refer to as the edge memory effect.

The TEV data of metabolically active mitochondria (Figure 5c) also reveal a distinct clustering of outliers at very low values (green markers in Figure 5c). A comparison of the corresponding average diameter (460 ± 33 nm, calculated as the diameter of spheres with equivalent volume) with that of the remaining mitochondria (1644 ± 88 nm, blue markers in Figure 5c) suggests that these outliers originate from much smaller objects. We assign them to submitochondrial particles [36]. The obtained diameter of metabolically active mitochondria (1.65 μm) aligns well with values reported in the literature [1,8]. A zoom into the outlier region in Figure 5c is provided in the Supporting Information File 1, Section S3, Figure S9. Notably, six out of eight mitochondria isolated using the mechanochemical method fall into this region. It is plausible that this isolation technique, which involves temperature shocks and shear forces, leads to a higher fraction of submitochondrial particles [36]. Assuming that the edge memory effect is specific to mitochondria and cannot be replicated by submitochondrial particles, the TEV for such submitochondrial particles should remain small and primarily influenced by instrumental factors. Furthermore, its time-dependent behaviour should resemble that of metabolically inactive objects. This hypothesis is validated in the subsequent analysis.

Figure 6a shows a difference image of a fixed mitochondrion and Figure 6b the TEV as a function of time *t*_P_ for fixed mitochondria. The data show no systematic time dependence, and the magnitude of the TEV is comparable to that observed for submitochondrial particles (green markers in Figure 5c). This observation further supports the utility of the TEV as a viability indicator.

The uneven edge distribution across the mitochondria is striking.

### Height intermittency

**Figure 7:**
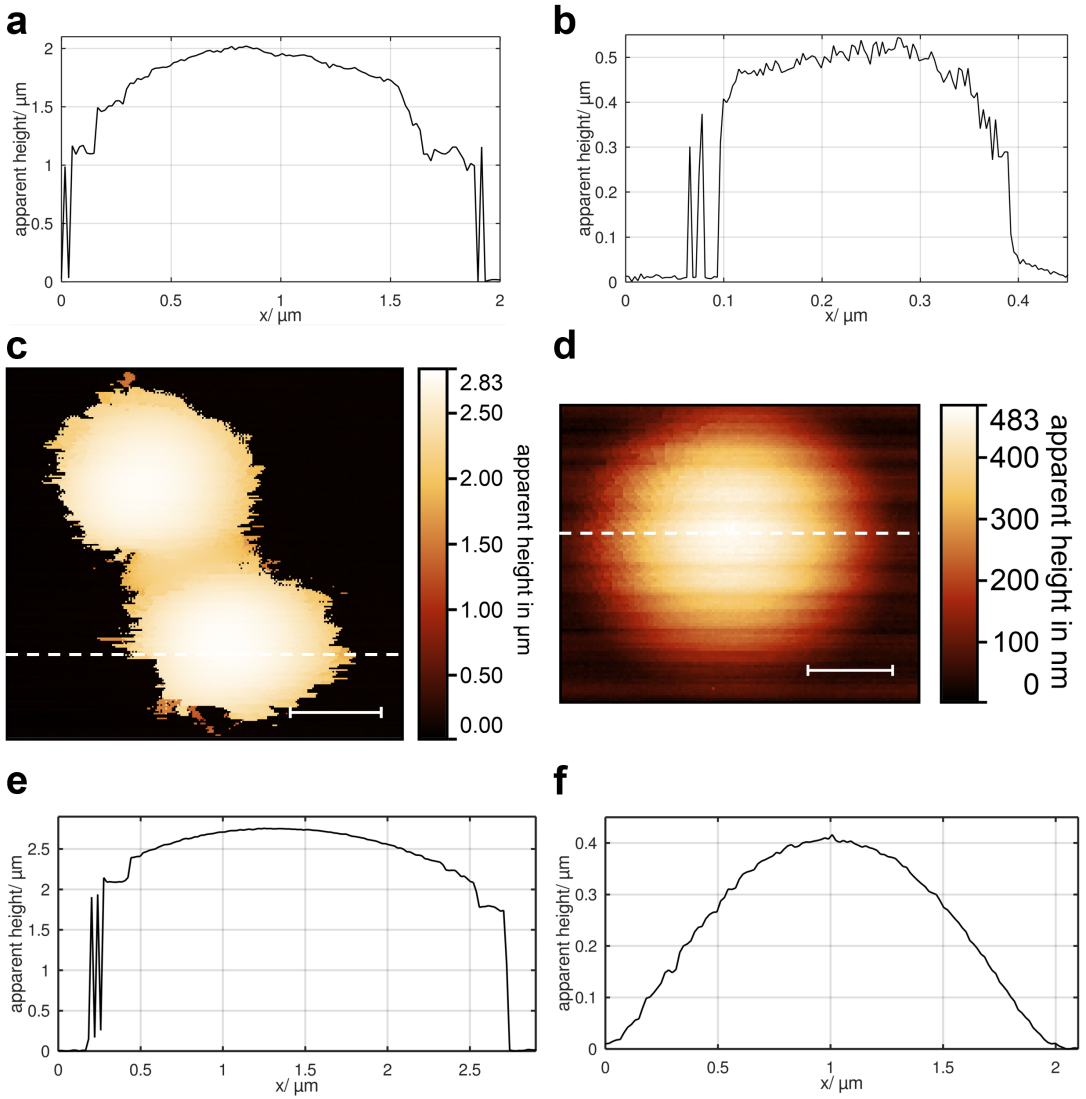
Measurements illustrating the intermittency effect, where dashed lines show the location of line profile. (a) Exemplary line profile of Figure 3a. (b) Exemplary line profile of Figure 3b; The line profile on the edges of the mitochondria drops repeatedly from mitochondrial to nearly substrate height. (c) False-colour topography of 3 μm microspheres, 256 × 256 pixels, *t* = 150 min. (d) False-colour topography of a 1 μm microsphere, 128 × 128 pixels, *t* = 15 min. (e) Exemplary line profile of (c); The line profile on the edges of the microspheres drops abruptly to nearly substrate height, which was observed repeatedly. (f) Exemplary line profile of (d); Here no intermittency is visible because of the more cell-like shape. Scale bars: (c) 1 μm; (d) 500 nm.

As demonstrated in Figure 3a,b,e,f as well as in Figure 4a, occasional abrupt height changes, referred to as height intermittency, are observed, primarily near the edges of mitochondria. These changes exhibit largely stochastic characteristics. In certain regions, they show an alternating pattern, fluctuating between large and small *z*-values. Exemplary line scans for two mitochondria with different *t*_P_ are shown in Figure 7a,b, corresponding to the mitochondria in Figure 3a,b. The *z*-values in these line scans span the full height range, from zero (indicating the substrate level, corresponding to the temporary absence of the mitochondrion at that position) to the maximum height of the mitochondrion. Interestingly, this height intermittency also occurs at positions several hundred nanometers away from the mitochondrion’s edge (see Supporting Information File 1, Section S4). To determine whether the height intermittency is specific to SICM topographies of metabolically active and fixed mitochondria, we compare these results with SICM topographies of polystyrene microspheres (non-living objects with nearly the same geometry). Given the wide variation in mitochondrial diameters (0.2–2.6 μm), we use two model objects for comparison, namely, microspheres with diameters of 1 and 3 μm. For 3 μm microspheres (Figure 7c,e), the same behaviour is observed qualitatively. However, 1 μm microspheres (Figure 7d,f) do not exhibit the intermittency effect. Because of the immobilisation protocol using acetone vapour, the 1 μm microspheres “melted” and adopted a more cell-like shape.

## Discussion

### SICM topographies and mitochondrial characteristics

The SICM topographies reflect the well-documented diversity of mitochondrial shapes and represent a significant advance in imaging metabolically active organelles in their native environment with nanometer-scale resolution. The outer mitochondrial membrane appears largely “featureless” and smooth in these measurements. No detectable signatures of porins or other integral membrane proteins, which are present in high density within the outer membrane, are observed. This absence can be attributed to membrane fluctuations in living and metabolically active systems, which exhibit amplitudes of several 10 nm. See Supporting Information File 1, Section S5, for more information. These fluctuations obscure the detection of smaller proteins. Identifying proteins on or within membranes would require advanced mathematical extraction techniques, such as deep learning-based analysis. Throughout the SICM measurements, no appreciable changes in the shape or undulation amplitude of the mitochondria were observed. This stability contrasts with the structural alterations reported with more violent techniques, such as deformability cytometry [37].

For mitochondria isolated using the nitrogen cavitation method, fixed mitochondria exhibit significantly smaller dimensions (Figure 4b). The reduction in size can be explained by the action of the fixative, formaldehyde, which removes water from the mitochondria, causing shrinkage [38–39].

### Morphodynamics

The edge memory effect is a phenomenon closely tied to the morphodynamics of mitochondria and serves as an indicator of their viability. This effect does not manifest in fixed mitochondria, polystyrene microspheres, or living cells (MG-63 osteoblasts; see Supporting Information File 1, Section S3) and has not been reported in the literature for either dead or living systems. Thus so far, it appears unique to mitochondria.

Over time, the morphodynamic activity of mitochondria diminishes because of the absence of nutrients in the measurement medium. As nutrients are depleted and metabolic byproducts accumulate, the TEV(*t*_P_) approaches zero after *t*_P_ ≈ 18.8 h. This indicates that the edge memory effect reflects an active morphodynamic reaction to the SICM measurement, as passive effects, such as drift phenomena, cannot account for the observed decreasing trend or the uneven edge distribution across the mitochondrion.

To find a possible explanation for the edge memory effect, we will focus on a key feature of the SICM setup, that is, the nanopipette. Our hypothesis is that the mitochondria actively react morphodynamically to the nanopipette’s surface potential properties.

**Figure 8:**
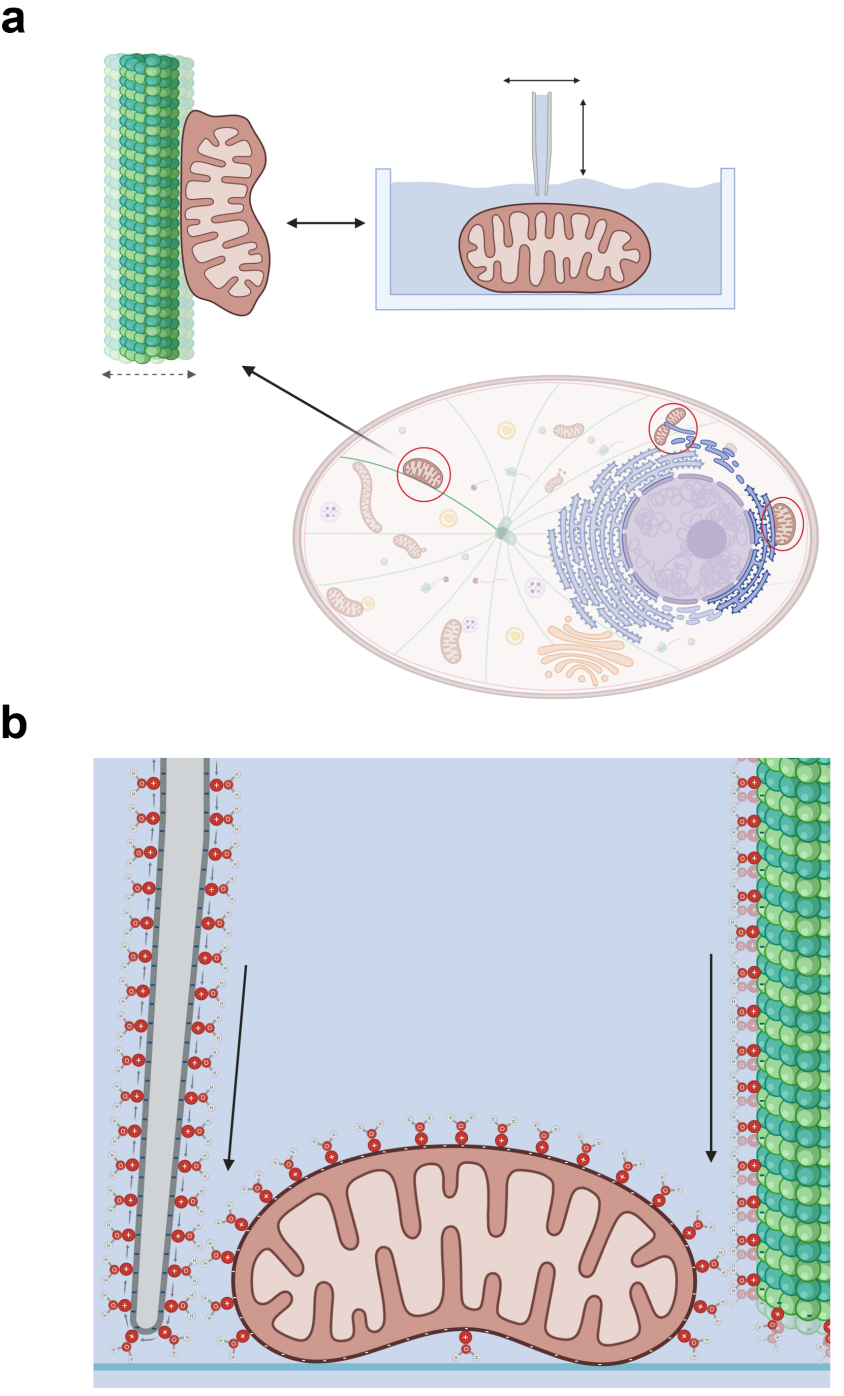
Schematic illustration of the morphodynamics of the mitochondrial network in cells and their hypothetical relation to the SICM measurement. (a) Illustration of a cell with organelles. The mitochondrial network acts with a wide range of cell organelles. The focus of the hypotheses is on the microtubules (green) as they are similar in shape and ζ-potential to the nanopipette. Microtubules induce a short-term change in shape and of the mitochondria through their “jittering”. Figure 8a was created in BioRender. Lieberwirth, E. (2025) https://BioRender.com/zs0463y. This content is not subject to CC BY 4.0. (b) Visualisation of the hypothesis on the similarity of the nanopipette and a microtubule. Nanopipette, mitochondrion, and microtubule have negative ζ-potentials. In a physiological medium, a cation layer forms on the surface (its hydration shell is represented by the water molecules). These cations on the glass of the pipette move through the pipette electrode into the pipette. A current flows, which generates an electric field (symbolised by the black arrow). The surface charges perform a relative movement due to the jittering of the microtubule. This also generates a current and an electric field from the point of observation of the mitochondrion (symbolised by the black arrow). Figure 8b was created in BioRender. Lieberwirth, E. (2025) https://BioRender.com/ate5331. This content is not subject to CC BY 4.0. Both illustrations not true to scale.

**Figure 9:**
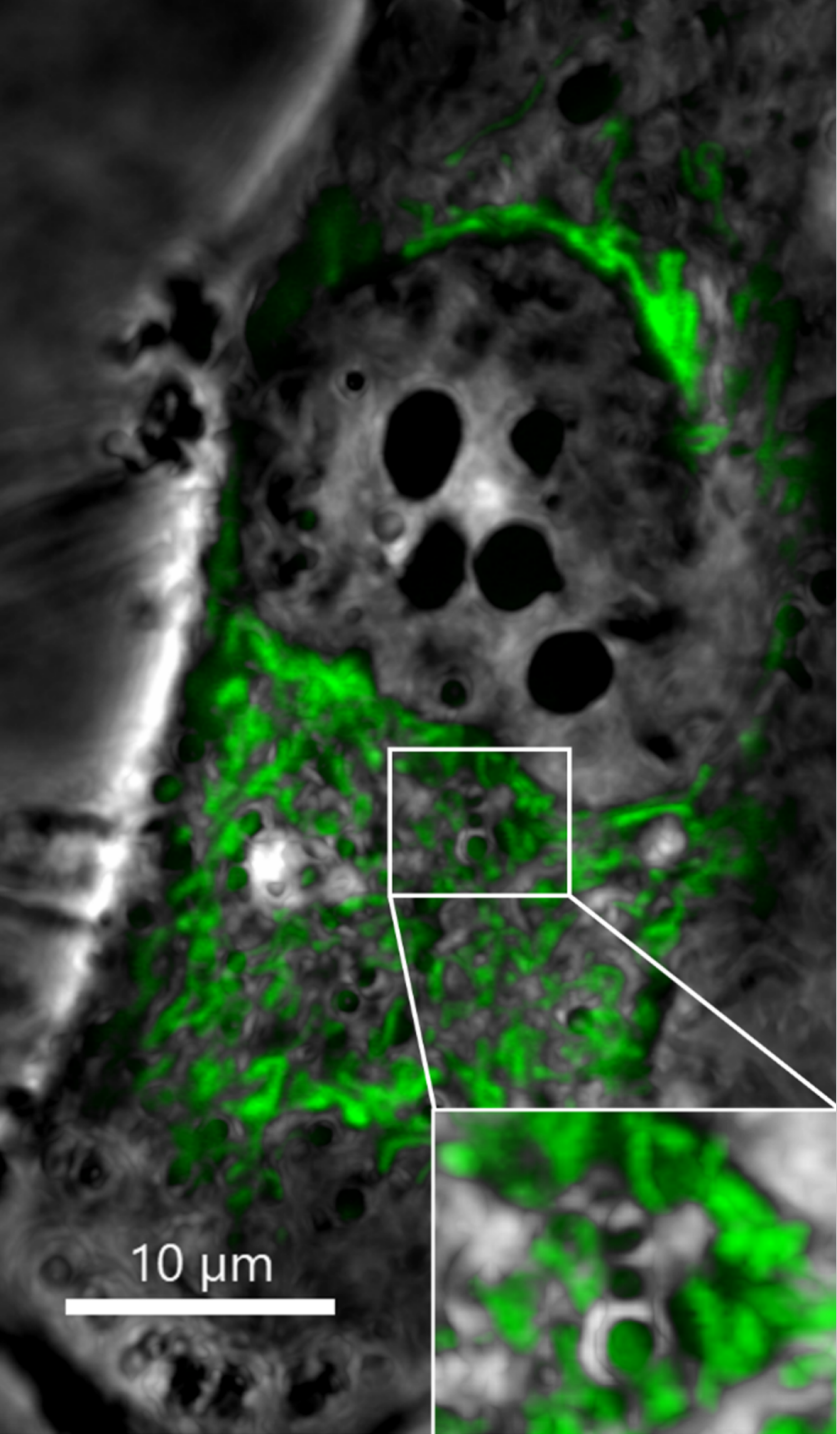
Mitochondrial network in an intact HeLa cell stained with MTG. Mitochondria (green) interact with several structures in the cytoplasm (visualised by phase contrast, grey) in particular with the endoplasmic reticulum in the vicinity of the cell nucleus (as shown in the inset). Scale bar: 10 μm.

In living cells, mitochondrial interactions with the cytoskeleton play a critical role in shaping and regulating mitochondrial function (Figure 8a and Figure 9). Among cytoskeletal components, microtubules are particularly notable for their ability to induce rapid mitochondrial shape changes. This is in contrast to F-actin and intermediate filaments, which primarily influence mitochondrial morphology for fission events [40–41], which were not observed in our experiments as they are unlikely in isolated mitochondria.

Remarkably, the geometric and mechanical properties of microtubules are comparable to those of the nanopipette. Microtubules are hollow structures with an opening diameter of 18 nm and a total diameter of 25–30 nm [42–43]. Their pushing force, in the range of 3–4 pN [44], is similar to the hydrodynamic forces exerted by the nanopipette.

Beyond mechanical similarities, microtubules and the nanopipette also share key “biophysical” properties. Both surfaces are strongly negatively charged, with ζ-potentials ranging from −30 to −60 mV [45]. Therefore, both exhibit a laterally mobile cation layer on their surfaces, which is responsible for ionic conductivity in physiological media (Figure 8b). Microtubules align along direct current electric field lines and can be vibrationally excited at megahertz frequencies by electric dipole antennas [46]. Other relevant properties include metabolically driven vibration modes [47], ferroelectricity [48], resonant ionic conductance, and memristive behaviour [49].

Mitochondrial morphodynamics respond to the proximity and orientation of microtubules. Non-spherical mitochondria near microtubules align their long axes with those of the microtubules, with response times of the order of seconds or less [6,41,50]. The binding process between microtubules and mitochondria is well known [40–41]. The precise mechanism by which mitochondria and microtubules locate each other remains unclear, but their interaction is too directional to be coincidental. We propose that this interaction is mediated by the ζ-potential of the microtubule, which signals to the mitochondria the intention to bind.

Given the structural and biophysical resemblance between the nanopipette and microtubules, it is plausible that the mitochondria misinterpret the nanopipette as a microtubule. As electric fields (and therefore the electric field originating from the ζ-potential, too) in physiological media are attenuated over short distances [27,51], a morphodynamic response would only occur when the nanopipette is within a few pipette diameters of the mitochondrion. The edge memory effect could thus represent a preemptive morphodynamic reaction by mitochondria anticipating contact with a microtubule.

The nanopipette remains close to the edge of the mitochondrion for approx. hundred milliseconds per scan line. This duration may be sufficient to elicit a morphodynamic response, as typical shape fluctuation rates are on the order of 75 ms^−1^ [41].

The asymmetry of the edge memory effect (evident in the variation of edge widths and the uneven edge distribution across the mitochondrion) is particularly striking. This suggests that mitochondria possess a form of “memory” for the nanopipette’s previous positions. Obviously this “memory” is not neurological in nature. It could result from metabolic processes or localised changes in the membrane induced by the nanopipette’s ζ-potential. Alternatively, a form of “passive memory” could arise from charge redistribution in response to the ζ-potential. For more information regarding the connection of ζ-potential and membrane fluctuations, refer to Supporting Information File 1, Section S5.

### Insufficient immobilisation causes height intermittency

The observed height intermittency effect originates from the combination of spherical sample geometry and insufficient immobilisation. While cells adhere to suitable substrates, often facilitated by membrane proteins that increase the contact area, mitochondria lack this ability. SICM studies of cells typically do not show displacement caused by the nanopipette, as their adhesion to the substrate prevents movement. In contrast, mitochondria, due to their spherical shape, make only minimal contact with the immobilising substrate. As a result, they may “tilt” when the nanopipette approaches too quickly, then relax back to their original position when the pipette moves away. This movement is likely driven by residual direct interaction forces or indirect hydrodynamic forces induced by the pipette’s hopping motion. Such tilting and subsequent relaxation can occur only during the pipette’s approach to or retraction from the mitochondrion. Additionally, the steep slope of the spherical mitochondria may cause the nanopipette to “bend” laterally around the sample [52–53]. The glass used for such fine nanopipettes is inherently soft and susceptible to bending when encountering sharp gradients.

The hypothesis of insufficient immobilisation is supported by the result that height intermittency occurs with fixed mitochondria and with 3 μm microspheres treated with acetone (non-living systems with similar geometry). The effect does not occur with 1 μm microspheres treated with acetone vaporisation. In this case, the microspheres’ shape becomes more cell-like, reducing the steep slope and thus preventing the intermittency effect.

A convolution effect, similar to the tip–sample convolution known from AFM, cannot account for the observed intermittency effect. The nanopipette tip exhibits an extremely high aspect ratio, in contrast to conventional AFM cantilever tips. A surface feature to trace the nanopipette must have a higher aspect ratio. The assumption that similarly long (hundreds of nanometers) and rigid surface structures could transiently act as a “tip” during imaging (an essential condition for convolution artifacts) is not plausible, particularly when imaging soft biological materials.

## Conclusion

Metabolically active mitochondria were extracted from HeLa cells, and their membrane topography and dynamics were studied using SICM. By employing a simple immobilisation protocol, we successfully examined the topography of metabolising mitochondria with a minimally interacting SPM method, demonstrating the diversity of mitochondrial shapes.

Two novel effects were observed:

1. The edge memory effect: This effect decreases linearly over time elapsed since preparation, suggesting a link to the mitochondrion’s metabolic activity, which diminishes over time because of nutrient depletion and increasing medium contamination. This appears to be an active mitochondrial response to the measurement process. We propose that the mitochondrion’s morphodynamic reaction is driven by its misinterpretation of the nanopipette as a microtubule due to shared physical properties, with the electric field originating from the ζ-potential acting as the mediating stimulus.
2. The intermittency effect: This phenomenon is attributed to the geometry of the mitochondria, which are only weakly in contact with the substrate due to their spherical or ellipsoidal shapes. Additionally, the lack of adhesion proteins results in weak immobilisation, allowing mitochondria to “tilt away” during the measurement process, rendering them transiently undetectable.

We demonstrated that SICM is a suitable technique for measuring metabolically active organelles with lateral resolutions of approximately 60–150 nm and a vertical resolution of about 1 nm. This opens new avenues for studying the behaviour and properties of isolated, metabolically active organelles at nanometer-scale resolution.

The identification of the edge memory effect provides a valuable marker for assessing mitochondrial viability during topography measurement as this effect is exclusively observed on metabolically active mitochondria. The TEV decreases with the time elapsed since the preparation.

Further verification of our hypothesis regarding the edge memory effect could involve systematic variations in the pipette movement scheme, such as transitioning static acquisition mode without hopping. It could be of interest to examine whether mitochondria isolated from primary tissue also exhibit the edge memory effect. Additionally, biochemical approaches, such as treating HeLa cells with oligomycin before mitochondrial isolation or manipulating specific proteins of the outer mitochondrial membrane, may shed light on the mechanisms underlying this phenomenon.

## Experimental

### Cell culture, isolation protocol, and viability measurements

HeLa cells [54–55] (CCL™), obtained from ATTC, were cultured and harvested in Dulbecco’s modified eagle medium (DMEM) at 37 °C in a 5% CO_2_ atmosphere. After harvesting, the cells were counted, requiring approximately 10^6^ to 10^7^ cells per milliliter per isolation pass, obtaining a satisfactory mitochondrial yield.

Mitochondria were isolated using the Mitochondria Isolation Kit for Cultured Cells (Abcam, UK), following the manufacturer’s instructions [56].

As a second isolation protocol, nitrogen cavitation was performed as described by Younis and coworkers [34]. This method is faster, gentler on mitochondria, and its components can be adapted for use with primary tissues. Nitrogen, which can permeate cell membranes but not the mitochondrial outer membrane, was used to disrupt the cells. Mechanical shear forces associated with the Dounce homogeniser, as well as the stress from freezing and thawing, were avoided to minimise the formation of submitochondrial particles [36].

Regardless of the isolation method, mitochondria were transported on ice [56]. For storage purposes, the mitochondrial fractions were aliquoted into 150 μL portions and frozen at −80 °C in Krebs–Ringer medium.

For optical microscopy, mitochondrial fractions were stained with 100 nmol·L^−1^ MitoTracker™ Green FM (Molecular Probes Invitrogen, Thermo Fisher Scientific Inc., USA) for 30 min at 37 °C, followed by staining with 5 mmol·L^−1^ TMRE (Molecular Probes Invitrogen Detection Technologies, Thermo Fisher Scientific Inc., USA) for 30 min at 37 °C. The samples were analysed using a Fluoview FV10i confocal microscope (Olympus, Germany).

Oxygen consumption was measured using an optode sensor-based system (Unisense, Denmark) as described in Schultz and coworkers [21]. Mitochondrial fractions of approximately 1 μg were investigated in bicarbonate-buffered Krebs–Ringer medium at 37 °C. Western blot analyses of 5–15 μg mitochondrial fraction isolated by nitrogen cavitation or mechanochemical method were performed as described in [21]. The following primary antibodies were used: anti-VDAC (1:500) (OriGene Technologies, Inc., USA), anti-GAPDH (1:1000) (Cell Signaling Technology, USA), and Total OXPHOS Rodent WB Antibody Cocktail (1:250) (Abcam, UK).

### Immobilisation protocol of metabolically active mitochondria and reference structures

Mitochondria suspended in Krebs–Ringer medium in a 1.5 mL Eppendorf tube were thawed at room temperature, if necessary. The tube was filled with Krebs–Ringer medium (approx. 1.4 mL), mixed, and 200 μL of the suspension was added to the bioinert cover slip of a “35 mm imaging dish with a polymer coverslip bottom and low walls” (Cat. No.: 80136, ibidi). After an incubation time of 10 min at room temperature to allow the mitochondria to settle on the coverslip, the excess medium was carefully removed. The imaging dish was gently rinsed with approx. 1 mL Krebs–Ringer medium and carefully refilled with 3 mL of fresh medium. It is important to add the medium gently, as the immobilisation of the mitochondria is very weak compared to cell adhesion. Excessively rapid addition of the medium risks washing the mitochondria away. The open imaging dish is transferred to the SICM.

To correlate observed effects with mitochondrial metabolic activity, fixed mitochondria were also investigated. For fixation, the mitochondria were incubated for 48 h in the imaging dish with 1 mL of 4% formaldehyde solution. The solution was subsequently removed, and the imaging dish was rinsed gently with 2 mL Krebs–Ringer medium before being refilled with 3 mL of fresh medium.

For comparison, reference measurements were performed on similarly shaped polystyrene microspheres with diameters of 1 and 3 μm. 100 μL of the microsphere ethanol solution (1:10 for 3 μm microspheres, 1:100 for 1 μm microspheres) was transferred to the imaging dish and left to dry, allowing the ethanol to evaporate. As immobilisation was insufficient, the samples were exposed to acetone vapour for 30 min at room temperature in air. The 3 μm polystyrene microspheres retained their approximate round shape, while the 1 μm polystyrene microspheres “melted”, adopting a morphology similar to adhered cells. The imaging dish was carefully filled with Krebs–Ringer medium. For detailed information, refer to Supporting Information File 1, Section S6.

The imaging dish was characterised using a AFM, see Supporting Information File 1, Section S7, Figure S19. The bioinert and hydrophilic polymer surface exhibited height variations up to 3 nm.

### Scanning ion conductance microscopy

Mitochondria were examined using a SICM, a SPM technique employing an ion current through a nanopipette as quantity to be measured by a feedback loop. This current is generated by applying a voltage between two Ag/AgCl electrodes, one located in an electrolyte bath containing the non-conductive sample and the other within the nanopipette (Supporting Information File 1, Section S7, Figure S20). When the nanopipette is gently approached vertically towards the sample, within immediate distance to its surface, the ion current decreases as the ion delivering volume between the nanopipette opening and the sample surface is reduced. This current drop is used by the feedback loop, which regulates the pipette’s height to maintain a constant distance from the sample surface. By mapping the pipette’s height pointwise, the sample’s topography is obtained [27–28].

Measurements were performed using a NX-Bio (Park Systems Corp., Korea) in hopping mode. In this mode, the pipette is gently approached to the surface at each pixel, and upon detecting a preset current drop of 2% relative to the standby current, it retracts to a defined height (hopping height) [27,35]. In our measurements, the standby current was approximately 1 nA by applying a voltage of 0.15–0.40 V between the bath electrode and the nanopipette electrode, depending on the opening diameter of the nanopipette. The nanopipettes were fabricated using a P-2000 (Sutter Instrument, USA) from borosilicate capillaries with an outer diameter of 1 mm and an inner diameter of 0.58 mm. The resulting opening diameter of the pipettes ranged from 45 to 100 nm, depending on the pulling parameters. Detailed information about the pipette fabrication can be found in Supporting Information File 1, Section S8.

The key feature of the hopping mode is the hopping frequency, which defines the measurement speed by indicating a number of pixels scanned per second. The hopping frequency depends on the pixel number per edge and the hopping height. For detailed information, refer to Supporting Information File 1, Section S9.

In our experiments, the hopping frequency ranged from 18 Hz (high sample and hopping heights) to 146 Hz (low sample and hopping heights). The pixel resolution ranged from 128 × 128 pixels to a maximum of 1024 × 1024 pixels, with scan ranges varying from 0.25 μm^2^ to 100 μm^2^. Each image line in the fast scan direction was scanned in both forward and backward directions, producing two images per measurement, that is, one forward and one backward scan.

The *z*-resolution of SICM is approximately 1 nm, while the lateral resolution is determined by the pipette opening diameter, ranging from 60% to 150% of the pipette opening radius (approx. 60 and 150 nm, respectively) [35,57–58].

The method is particularly well suited for studying living or metabolically active structures, as samples remain immersed in a nutrient medium and are not physically contacted. Unlike AFM, SICM measurements exert no mechanical force directly on the sample, and the ion currents involved are minimal. Although electric fields diminish rapidly over short distances [27–28,51], the pipette exerts a hydrostatic pressure on the sample. This pressure, influenced by the pipette length, amounts to approximately 500 Pa in our case. Furthermore, fluid flux from the pipette can exert forces up to 400 pN. However, because of the small opening diameter of the pipettes used, these forces were estimated to be in the range of 1 pN [59]. The disadvantage of SICM is the long measurement time.

### Data processing

SICM measurements were analysed using the free software Gwyddion 2.60 [60]. Images were cropped as needed, followed by a scanline mismatch correction using the row alignment median if possible. If this was unsuitable, the row alignment median of differences was applied and subsequently the background was levelled using subtraction by the mean value plane. The minimum value was set as the zero point, and the images were colour-coded using the Gwyddion.net colour pattern. Difference images were colour-coded with a custom colour pattern, where the centre of the scale is white, positive heights are red, and negative heights are blue. Line profiles were always extracted in Gwyddion along the fast scanning direction (*x*-axis). The line profile data was read and processed in the free software Octave 9.2.0., as well as the bar graphs, histograms, and fits.

Shape analyses of the mitochondria were performed using ImageJ 1.54g. Under the ’Analyze’ menu, the ’Set Measurements’ option was used to select area and perimeter. The pixel dimensions of the image were converted to physical units via the ’Set Scale’ function. The perimeter of each mitochondrion was manually outlined using the polygon selection tool, and measurements were obtained using the ’Measure’ function.

## Supporting Information

Further information to mitochondrial morphodynamics and the measurement setup; Supporting information provides further mitochondrial topographies and difference images, and detailed information about the measurement setup and microspheres. The following sections are included: S1: SICM measurements of metabolically active and fixed isolated mitochondria; S2: Bimodal distribution of the diameter of metabolically active mitochondria; S3: Calculated difference images of a MG-63 osteoblast, metabolically active, isolated mitochondria, and a 3 μm microsphere, as well as further calculations to visualise the edge memory effect; S4: Additional observations at metabolically active mitochondria; S5: Connection of ζ-potential and membrane fluctuations; S6: SEM measurements of the polystyrene microspheres; S7: Details of the imaging dishes and SICM setup; S8: SEM measurements and other details of the nanopipettes; S9: Further information about the hopping mode at our SICM setup.

File 1 Additional experimental data.

## Data Availability

Data generated and analysed during this study is openly available in Zenodo at https://doi.org/10.5281/zenodo.10964930.
