# Supplementary material for: Shape, membrane morphology, and morphodynamic response of metabolically active human mitochondria revealed by scanning ion conductance microscopy
Source: Beilstein J Nanotechnol. 2025 Jun 30;16:951–67. doi: 10.3762/bjnano.16.73 (PMC12230334; doi:10.3762/bjnano.16.73)
Supplement: File 1 — Additional experimental data. [file Beilstein_J_Nanotechnol-16-951-s001.pdf]

BEILSTEIN JOURNAL OF NANOTECHNOLOGY

Supporting Information

for

Shape, membrane morphology, and morphodynamic response of metabolically active human mitochondria revealed by scanning ion conductance microscopy

Eric Lieberwirth, Anja Schaeper, Regina Lange, Ingo Barke, Simone Baltrusch and Sylvia Speller

Beilstein J. Nanotechnol. **2025,** *16,* 951–967. doi:10.3762/bjnano.16.73

Additional experimental data

License and Terms: This is a supporting information file under the terms of the Creative Commons Attribution License (https://creativecommons.org/licenses/by/4.0). Please note that the reuse, redistribution and reproduction in particular requires that the author(s) and source are credited and that individual graphics may be subject to special legal provisions.
The license is subject to the *Beilstein Journal of Nanotechnology* terms and conditions: (https://www.beilstein-journals.org/bjnano/terms)

S1 SICM measurements of metabolically active and fixed isolated mitochondria

In the following Figures S1–S4, the diversity of mitochondrial shapes is illustrated by further SICM measurements.

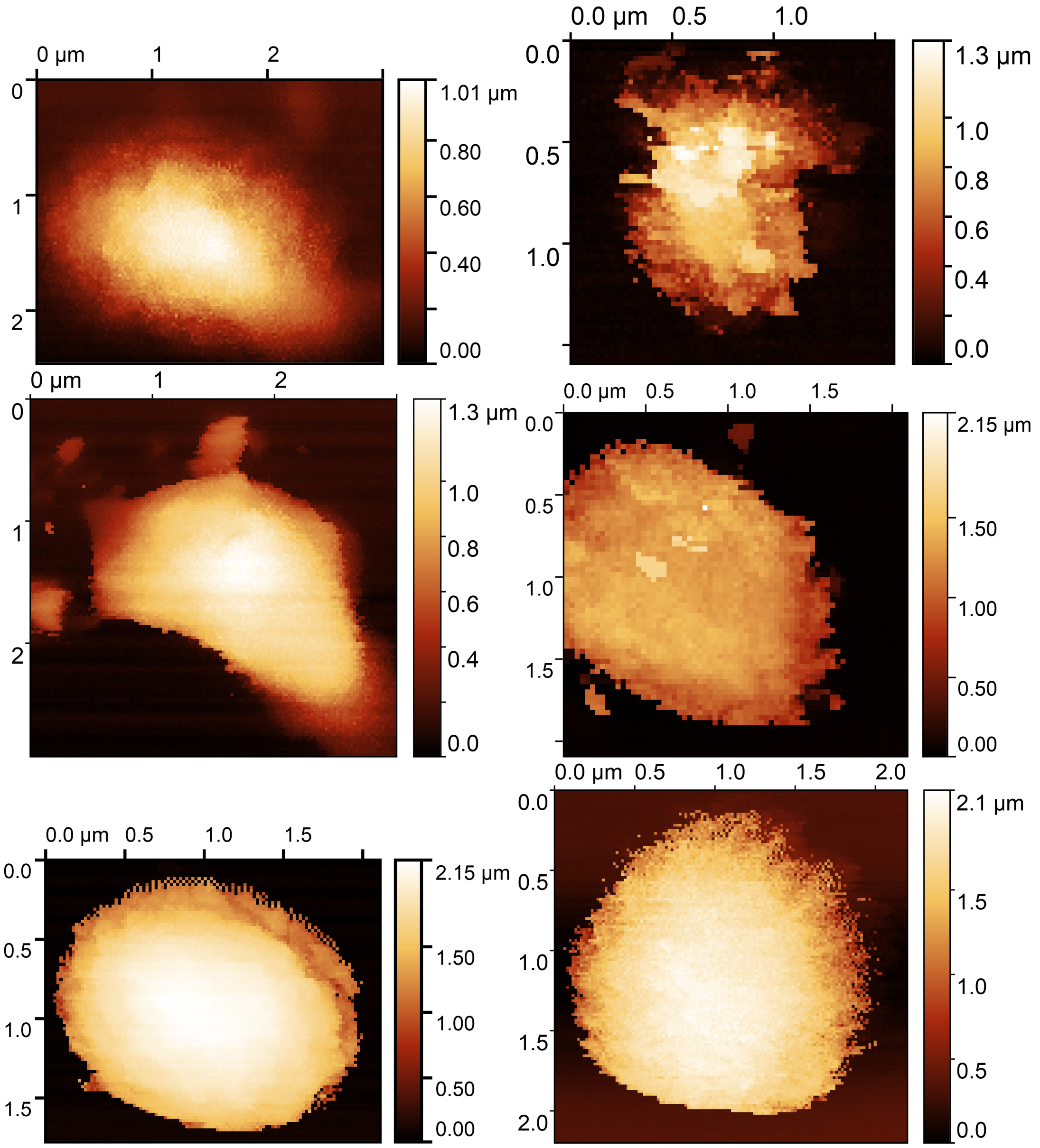

Figure S1: False colour topographies of mitochondria. First mitochondrion isolated by mechanochemical protocol. Others isolated by nitrogen cavitation. $128 \cdot 128$ pixels, measurement time $t \approx 30$ min.

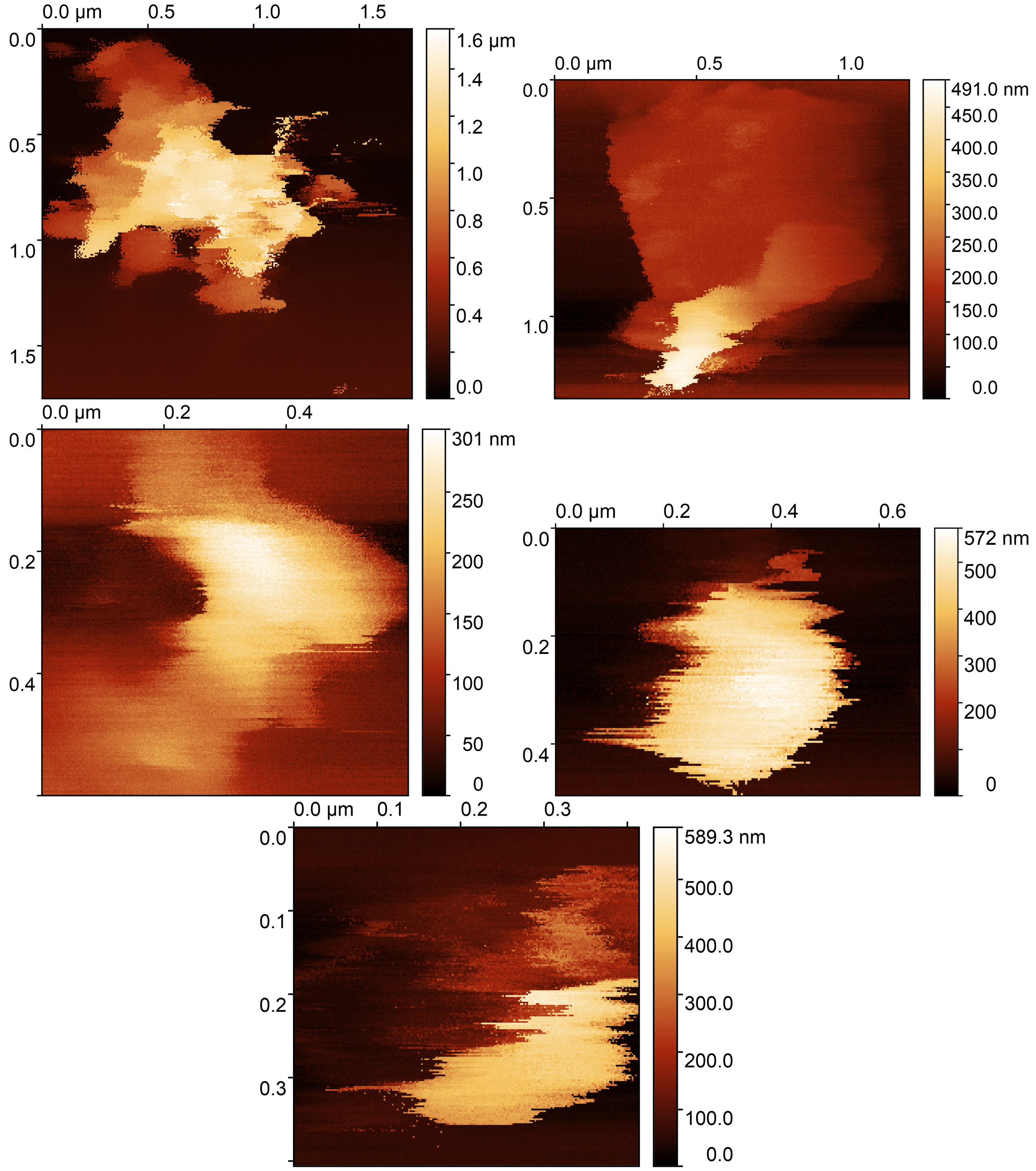

Figure S2: False colour topographies of mitochondria. Isolated by mechanochemical protocol. $256 \cdot 256$ pixels, $t \approx 60$ min.

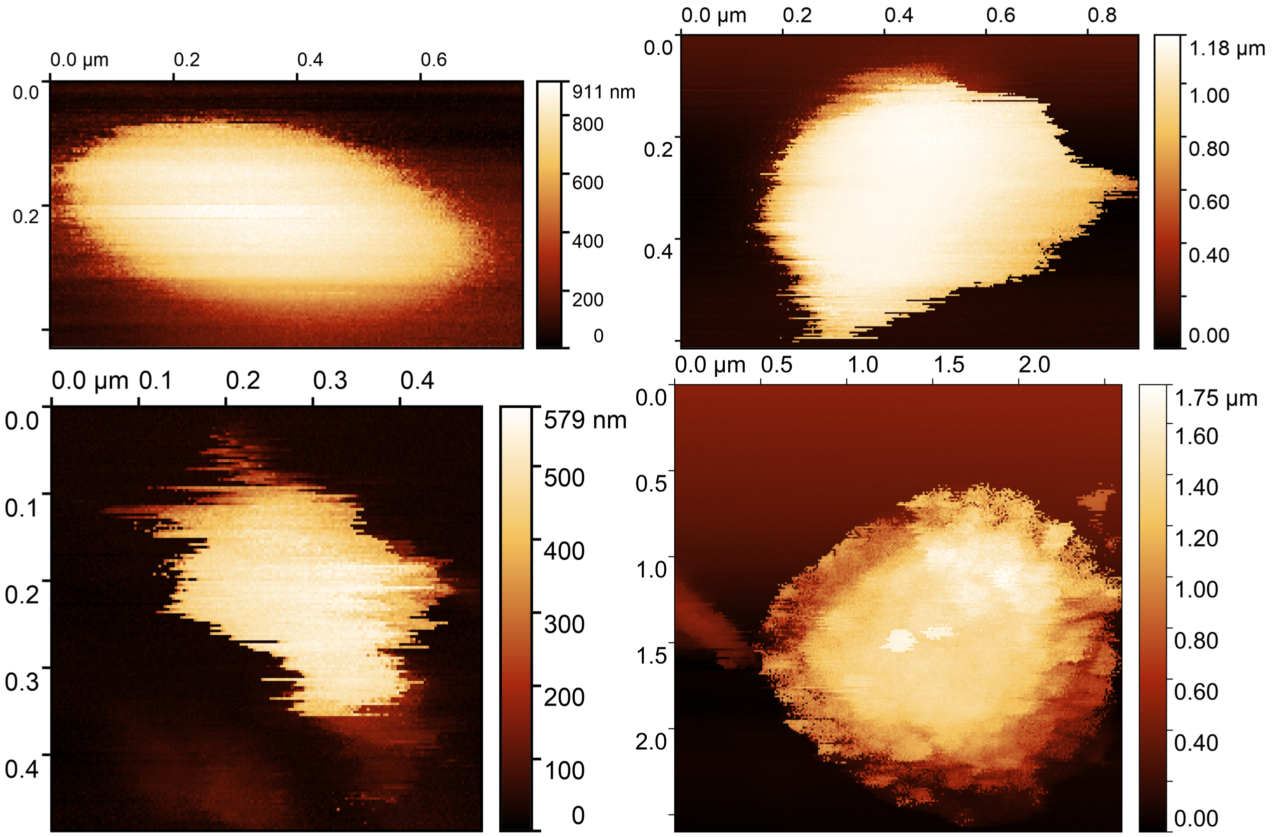

Figure S3: False colour topographies of mitochondria. Isolated by nitrogen cavitation. 256 · 256 pixels, $t \approx 60$ min.

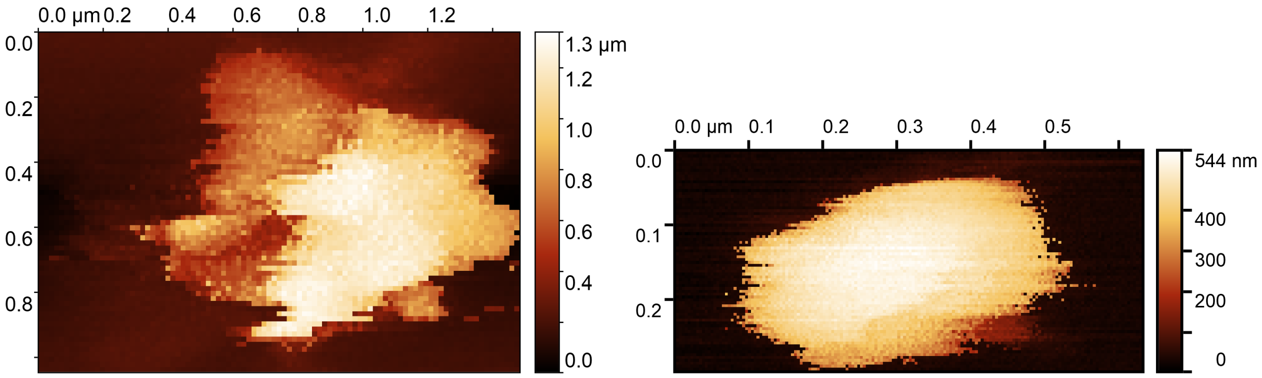

Figure S4: False colour topographies of fixed mitochondria. Isolated by mechanochemical protocol. first: 128 · 128 pixels, $t = 20$ min; second: 256 · 256 pixels, $t = 50$ min.

S2 Bimodal distribution of the diameter of metabolically active mitochondria

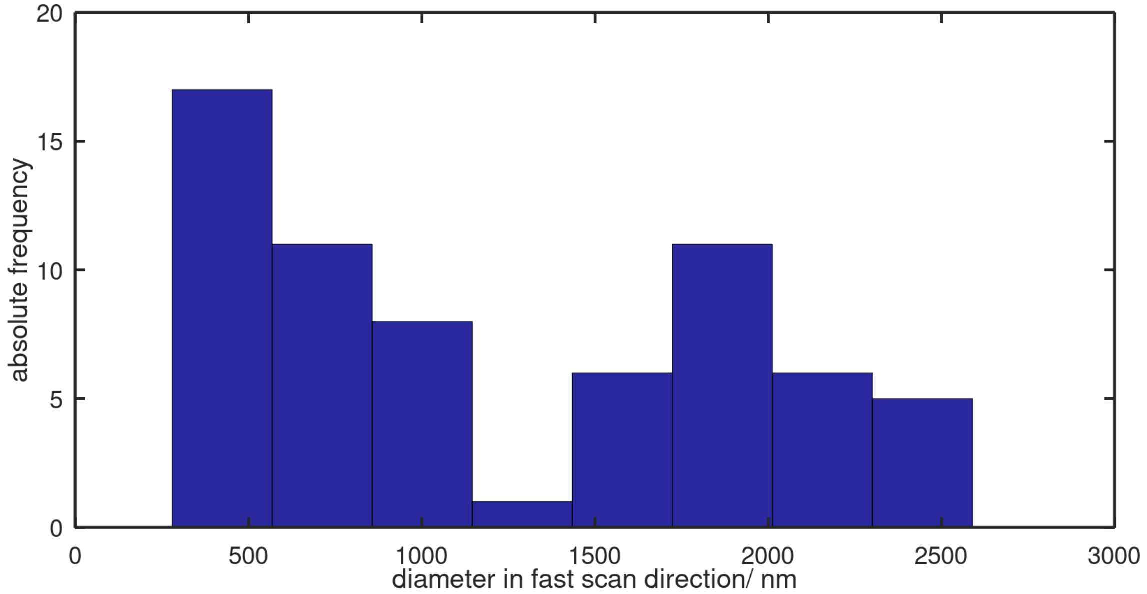

Figure S5: Histogram of the calculated mitochondrial diameters. The volume of each mitochondrion was derived from the measurements. Assuming an approximately spherical shape, the mitochondrial diameters were calculated.

S3 Calculated difference images of a MG-63 osteoblast, metabolically active, isolated mitochondria, a 3 µm microsphere and further calculations to visualise the edge memory effect

Since the SICM captures two images per measurement, differing only in the fast scan direction (left-to-right or right-to-left), difference images can be used to identify drift, morphodynamics and other phenomena. Drift-related effects and inaccuracies in the lateral positioning of the nanopipette typically manifest as an uniformly distributed blue or red edge around the object, which is not unusual. A representative example of drift in SPM is shown below using a difference image of an adherent but living MG-63 osteoblast (Figure S6). To further illustrate the distinction between SPM drift phenomena and the edge memory effect, additional difference images of metabolically active mitochondria are presented in Figure S7. In Figure S8, the edge memory effect is demonstrated for a single mitochondrion through a series of five consecutive measurements.

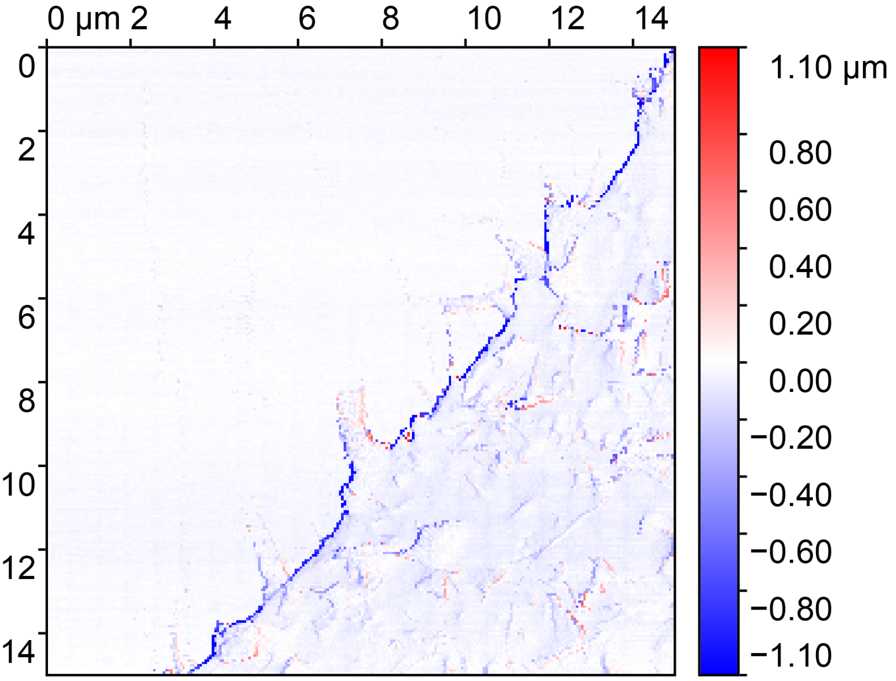

Figure S6: Difference image of a MG-63 osteoblast, which illustrates common SPM drift and hysteresis phenomena.

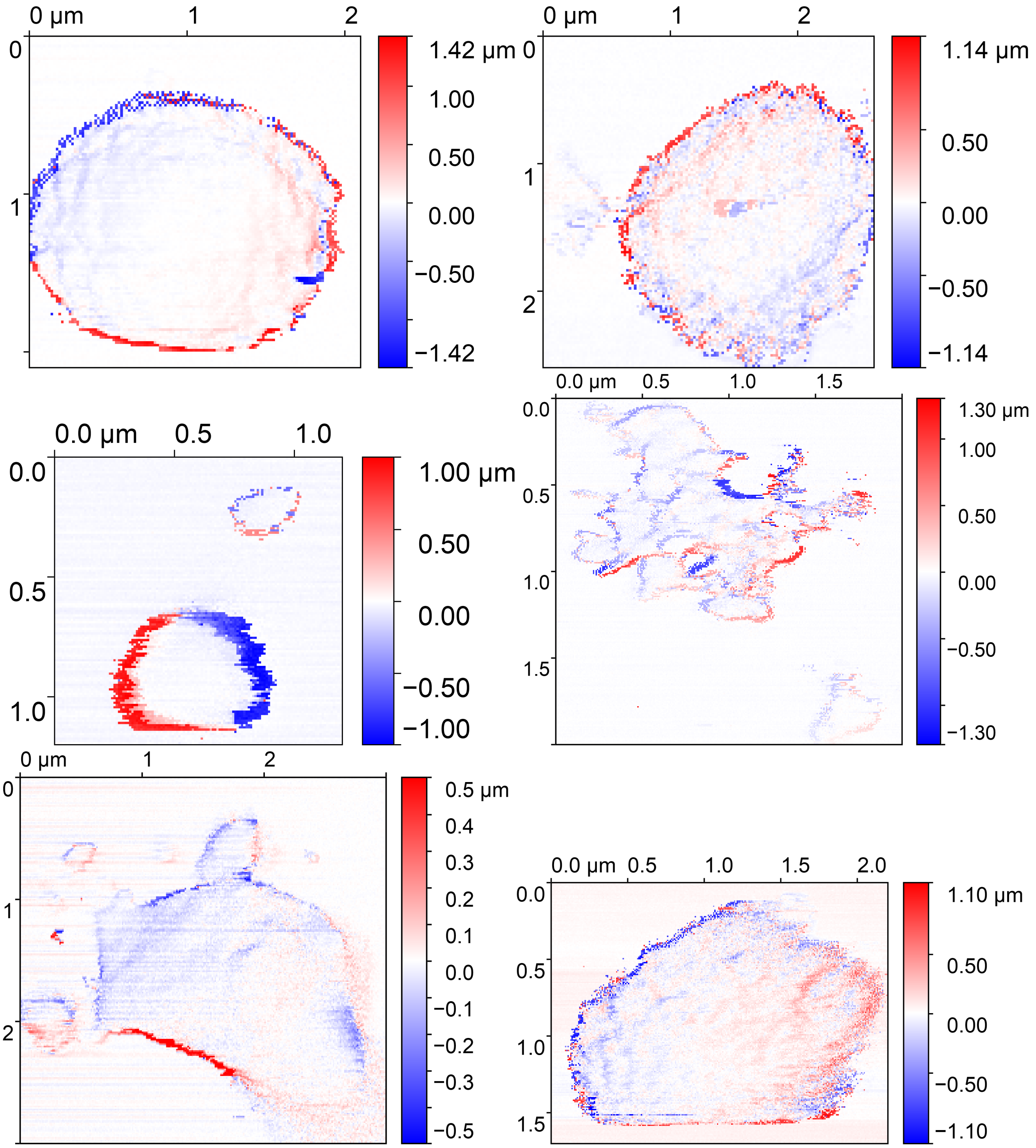

Figure S7: Difference images of various mitochondria, arranged with decreasing edge volumes from left to right and top to bottom. Note that the edge volume is calculated, not the edge width or edge area.

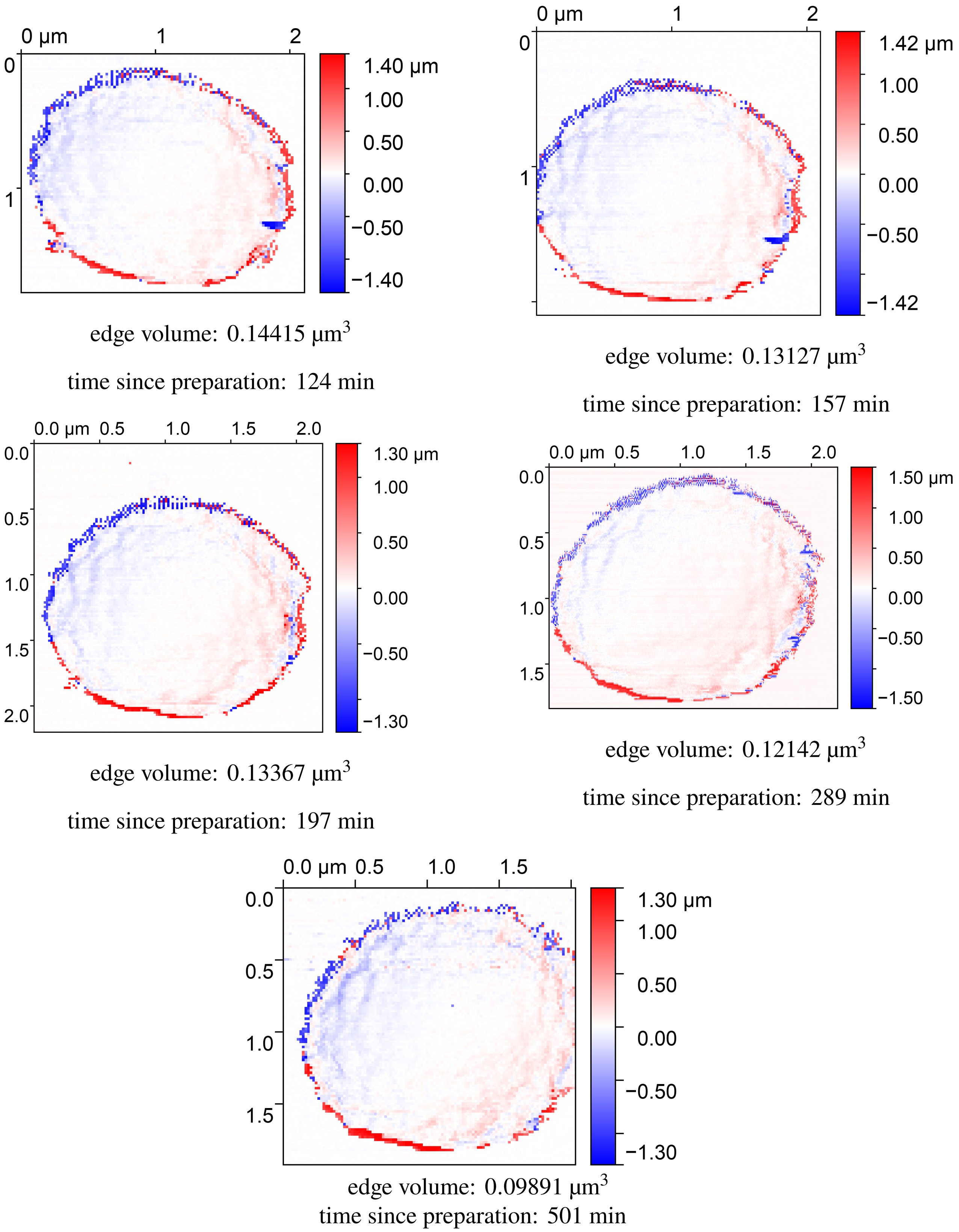

Figure S8: Difference images of a single mitochondrion with decreasing edge volumes and increasing time since preparation.

Figure S9 presents a zoom of Figure 5c to highlight the TEV of submitochondrial particles. The TEV is not time-dependent. Submitochondrial particles are clearly smaller and exhibit no edge memory effect. The TEV of submitochondrial particles is an order of magnitude smaller than that of metabolically active mitochondria.

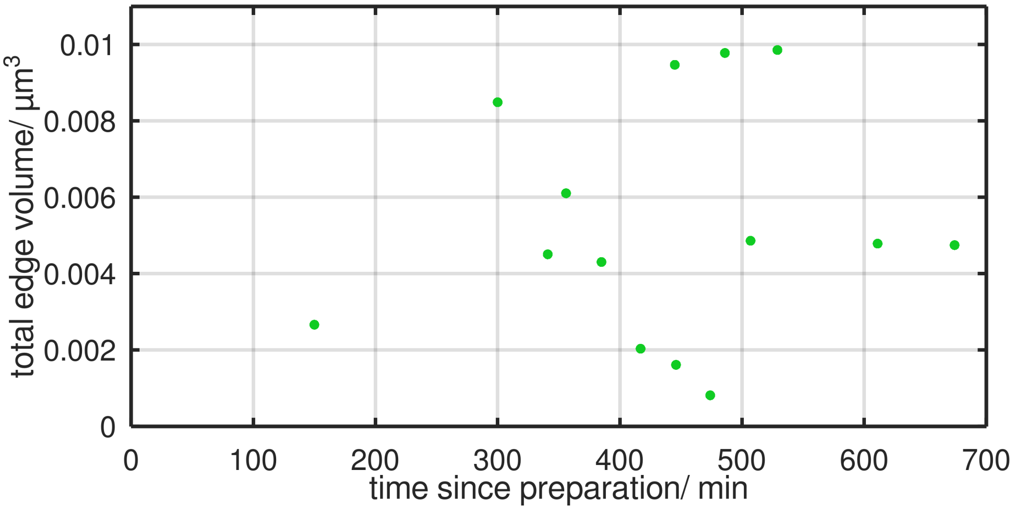

Figure S9: Graph depicting the TEV of submitochondrial particles as a function of time elapsed since the preparation. No correlation is observable between the data points. The TEVs of submitochondrial particles do not exhibit the edge memory effect.

Difference images for the microspheres are also possible (Figure S10). The TEV of the 3 µm microspheres is approx. ten times higher than that of metabolically active mitochondria as the microspheres have a volume that is 7 to 20 times larger. Consequently, the TEV normalised to the total volume of the microspheres is shown in Figure S11. The normalised TEV of the microspheres is in a range similar to that of the metabolically active mitochondria. However, the TEV of the microspheres does not exhibit any time dependence.

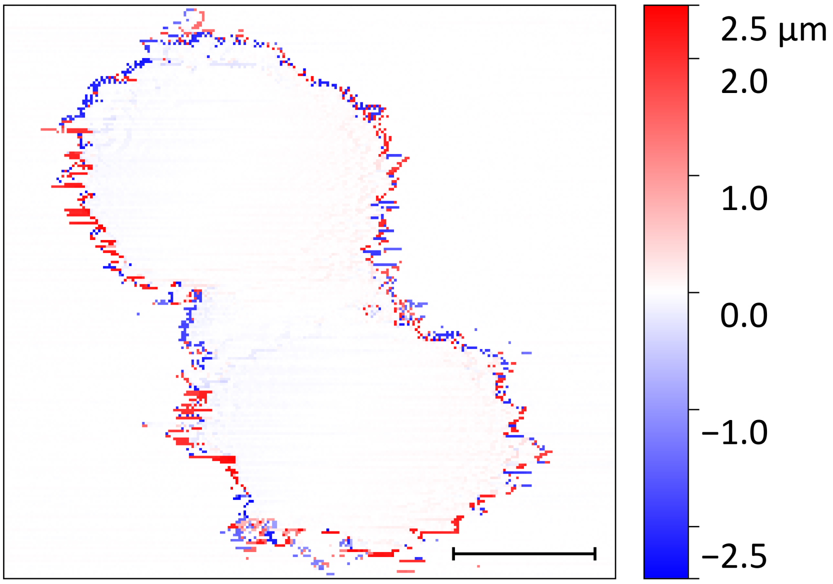

Figure S10: Difference image of a 3 µm microsphere, 256 · 256 pixels, t = 50 min, scale bar: 1 µm.

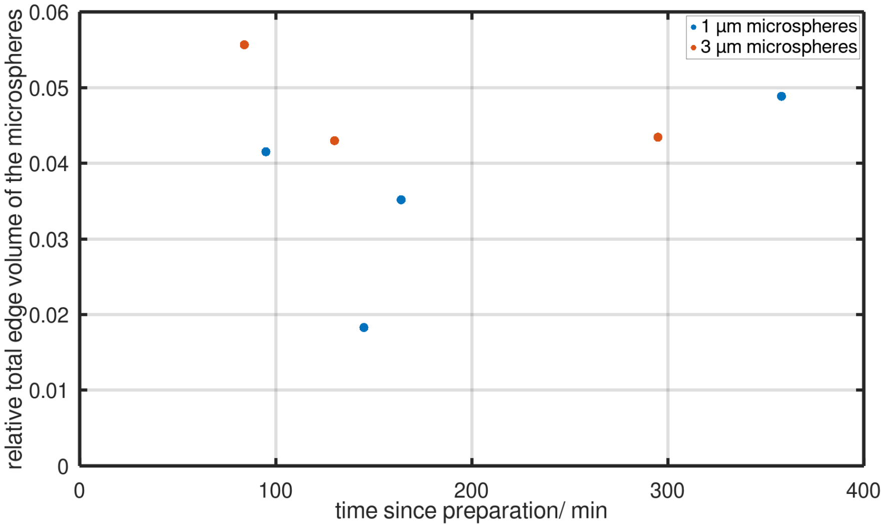

Figure S11: Graph showing the TEV normalised to the total microsphere volume on the time passed since preparation.

S4 Additional observations at metabolically active mitochondria

The intermittency effect is generally observed at the edges of mitochondrial topographies and is characterised by dark points at the organelle's borders. Occasionally, mitochondria exhibit transient presence events at locations distant from their main structure. This phenomenon occurs exclusively in mitochondria isolated using the mechanochemical protocol. An example is illustrated in Figure S12, where the mitochondrion terminates at $x = 1.2$ µm. However, at $x > 1.5$ µm (more than 300 nm away from the mitochondrion), two additional peaks with apparent heights of approximately 1.0 and 0.6 µm are observed. A plausible explanation for this behaviour is the generation of submitochondrial particles during the mechanochemical isolation process, which subsequently become immobilised at these locations.

A subset of mitochondria, particularly from the fraction isolated via the mechanochemical method, appears to have ruptured outer membranes. Such mitochondria progressively flatten and spread during the scanning process (Figure S13 left). When the nanopipette repeatedly scans over these ruptured mitochondria, a growing number of spikes (i.e., sudden height increases spanning only one or a few pixels, corresponding to fractions of a second) can be observed (Figure S13). These spikes have apparent heights ranging from 200 to 800 nm. A histogram of the apparent spike heights is provided in Figure S14.

One hypothesis is that the larger spikes correspond to mobile mitochondria loosely bound to the surface, diffusing and being transiently detected for only one or a few pixels. A more plausible explanation attributes these spikes to submitochondrial particles, which may form during mitochondrial isolation when subjected to shear forces, osmotic shocks, or cycles of freezing and thawing.

When the outer membrane ruptures, the inner membrane may invert, exposing the protein complexes involved in oxidative phosphorylation. This inversion can result in the formation of small, vesicle-like submitochondrial particles [Wilson, S. B.; Bonner, W. D., Jr. *Plant Physiol.* **1970**, *46*, 25–30. doi:10.1104/pp.46.1.25]. These particles may be only weakly immobilised or entirely free-floating due to their small size, which allows them to remain suspended in the medium. The hydrodynamic

forces generated by the nanopipette can cause passive movement of these particles. If they happen to drift in front of the approaching pipette, they are detected as transient spikes in the data.

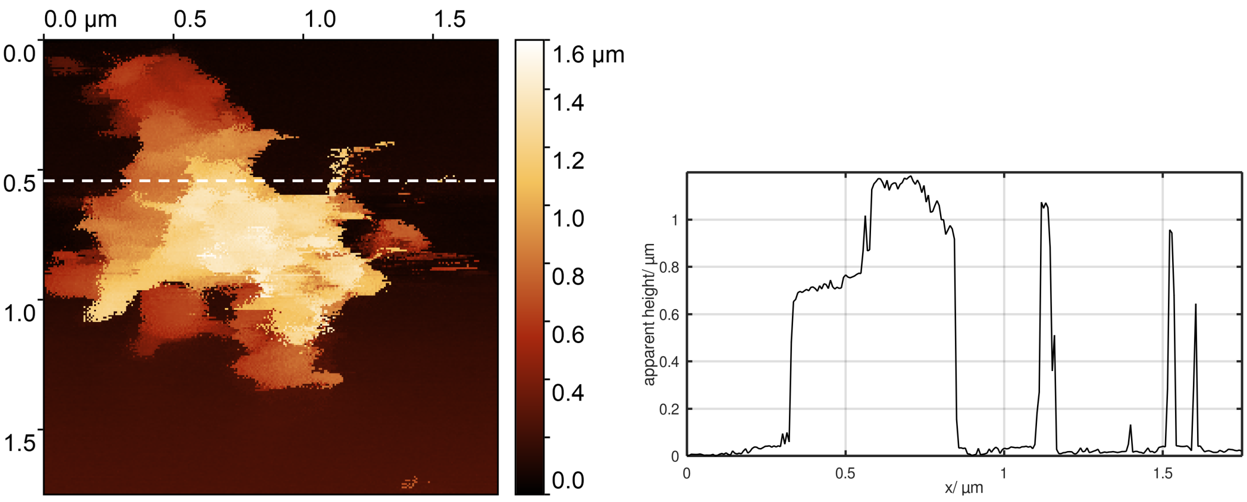

Figure S12: False colour topography of a mitochondrion (left). The line scan (right) showing the intermittency effect with far away transient presence events. Isolated by mechanochemical protocol. 256 · 256 pixels, t = 70 min.

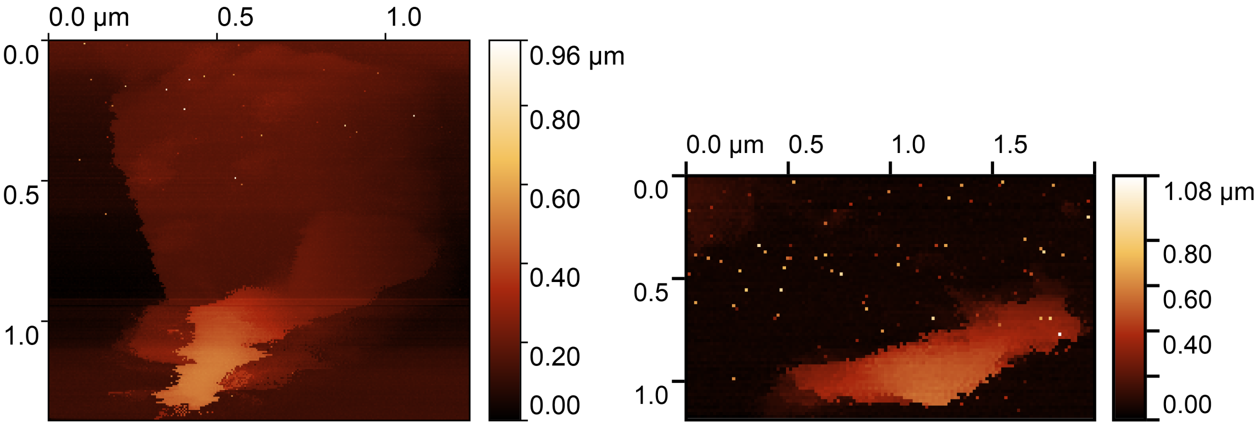

Figure S13: False colour topographies of two mitochondria with spikes. Isolated by mechanochemical protocol. left: 256 · 256 pixels, t = 50 min; right: 128 · 128 pixels, t = 20 min.

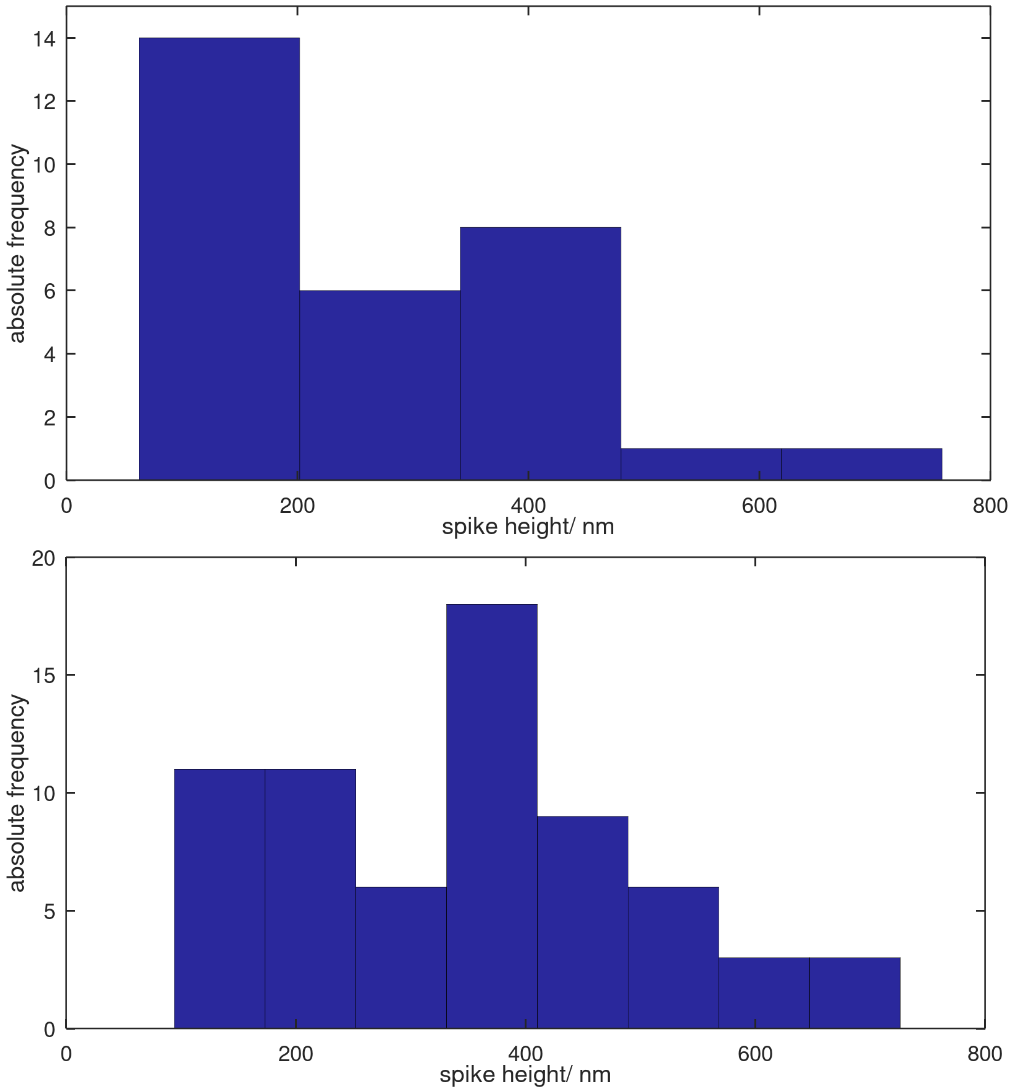

Figure S14: Histogram of the apparent spike heights of two mitochondria from Figure S13. The upper histogram shows the apparent spike heights from Figure S13 left. The lower histogram shows the apparent spike heights from Figure S13 right. The apparent spike heights are mostly in the range of 100–400 nm, which corresponds to the typical diameter of submitochondrial particles.

S5 Connection of ζ-potential and membrane fluctuations

We propose that the edge memory effect originates from the ζ-potential of the nanopipette, as the mitochondrion may misinterpret the nanopipette as a microtubule. In the following section, we examine the relationship between the ζ-potential and membrane fluctuations in more detail.

It is unlikely that the ζ-potential is completely screened, due to the short Debye length. However, hydrodynamic forces and friction can strip loosely bound ions from the pipette surface, thereby disrupting the screening layer. The resulting residual surface potential corresponds to the effective ζ-potential [Butt, H.-J.; Graf, K.; Kappl, M. *Physics and Chemistry of Interfaces*; Wiley-VCH Verlag & Co.: Weinheim, Germany, 2003; pp 72 and following].

In vitro, membrane fluctuations are responsible for generating local hydrodynamics. We assessed these fluctuations in mitochondria using SICM. When imaging at a fixed position or within a small frame (of the order of a few nanometers), SICM height traces follow the vertical displacement dynamics of the membrane. After applying drift correction and binning (applying a moving average filter with the window size of 100 and excluding the first 100 values), the root-mean-square displacement of the outer membrane $\left(\sqrt{< h^2 >}\right)$ was determined to be 19 nm at metabolically active mitochondria (Figure S15 top) and 7.4 nm (Figure S15 bottom) at fixed mitochondria. These values are comparable to those observed in whole cells, such as 21 and 9 nm for living and fixed osteoblasts, respectively [Voelkner, C.; Wendt, M; Lange, R.; Ulbrich, M.; Gruening, M.; Staehlke, S.; Nebe, B.; Barke, I.; Speller, S. *Beilstein J. Nanotechnol.* **2021**, *12*, 242–256. doi:10.3762/bjnano.12.20].

In fixed mitochondria, where metabolically driven membrane fluctuations are absent, the apparent vertical membrane stiffness $\left(k_{vertical} = \frac{k_B \cdot T}{<h^2>}\right)$ can be estimated using an equipartition-based approach. Notably, the measured height fluctuation amplitudes are typically only a few nanometers. As a result, unlike in force spectroscopy or suction experiments, which involve deeper membrane deformation, the derived apparent stiffness values reflect only the mechanical properties of the supported membrane itself, without contributions from underlying structures such as the cytoskeleton or cristae. The apparent vertical stiffness of the outer mitochondrial membrane was found to be approximately 11.3 $\frac{\mu\mathrm{N}}{\mathrm{m}}$, which is higher than that of living osteoblasts $\left(\approx 10\ \frac{\mu\mathrm{N}}{\mathrm{m}}\right)$ [Voelkner, C.; Wendt,

M; Lange, R.; Ulbrich, M.; Gruening, M.; Staehlke, S.; Nebe, B.; Barke, I.; Speller, S. *Beilstein J. Nanotechnol.* **2021**, *12*, 242–256. doi:10.3762/bjnano.12.20], but substantially lower than values obtained from suction-based $\left(9\ \frac{\mathrm{mN}}{\mathrm{m}}\right)$ [Wang, S.; Jiang, C.; Zhang, Y.; Chen, J.; Wang, B.; Chen, Q.; Long, M. *Cell. Mol. Bioeng.* **2008**, *1*, 67–74. doi:10.1007/s12195-008-0002-1] or microfluidic experiments [Komaragiri, Y.; Panhwar, M. H.; Fregin, B.; Jagirdar, G.; Wolke, C.; Spiegler, S.; Otto, O. *Biomicrofluidics* **2022**, *16*, 064101. doi:10.1063/5.0111581].

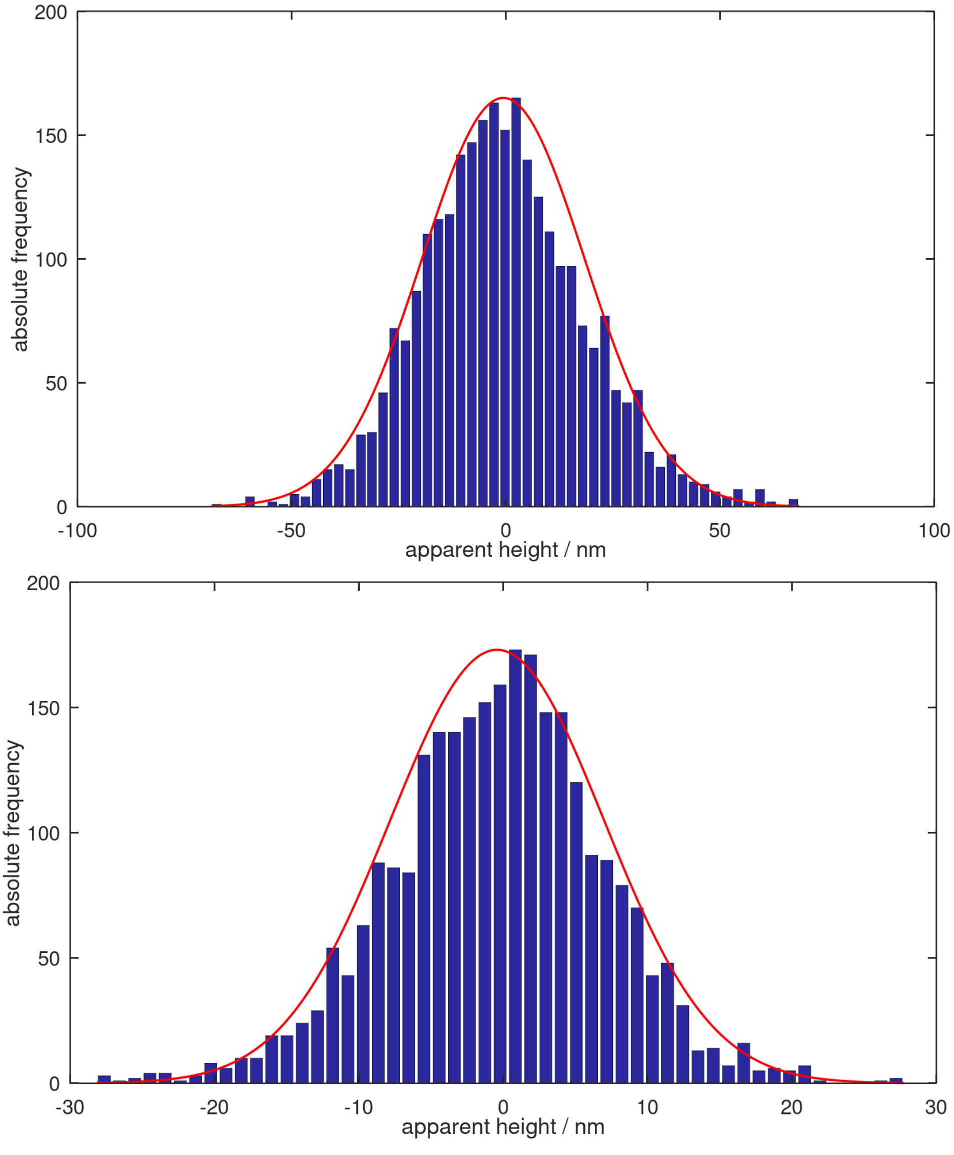

Figure S15: Histograms of the apparent heights of a point scan after approaching at the top of a mitochondrion with respective GAUSSIAN fit, top: metabolically active mitochondrion, bottom: fixed mitochondrion.

S6 SEM measurements of the polystyrene microspheres

Polystyrene microspheres (MICRO PARTICLES GMBH, Germany) with diameters of 1 and 3 µm were characterised using a scanning electron microscope (SEM, ZEISS EVO MA10, Germany). The SEM measurements provide a reference for comparison with the SICM results. Both sample types were prepared following the same protocol as those used for SICM analysis.

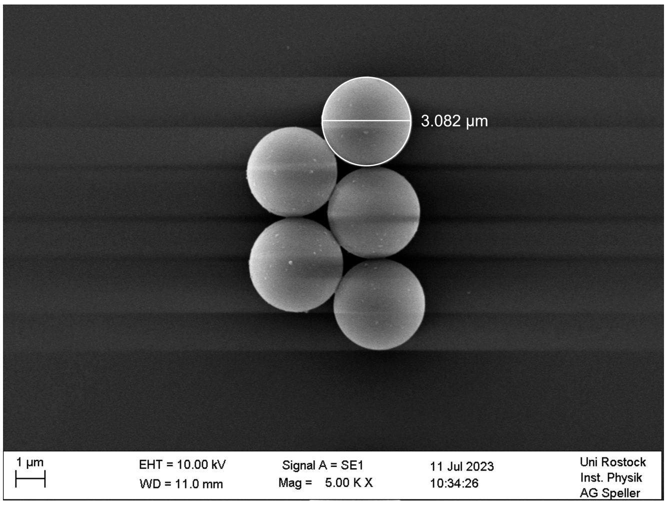

Figure S16: SEM measurement showing the 3 µm microspheres without acetone vaporisation.

The measured diameter of the 3 µm microspheres was within the specified tolerance range (3.082 µm, Figure S16). The projected area of these spheres forms a circle with numerical eccentricity $\varepsilon = 0$. Additionally, individual spheres within the conglomerate could be distinctly resolved as separate objects.

In contrast, 3 µm microspheres exposed to acetone vaporisation (Figure S17) appeared to be fused together by filaments. This can be attributed to the plastic-dissolving properties of acetone. During vaporisation, the surface of the spheres partially dissolves, leading to an increase in their contact areas. Once the vaporisation process is complete, the plastic hardens, forming adhesive-like bonds between the spheres. While the projected surface area remains approximately circular, it becomes more challenging to assess due to the fusion of adjacent spheres. Notably, the measured diameter increased to 3.2 µm, which exceeds the specified tolerance range. This increase is attributed to the "melting" effect caused by acetone vaporisation.

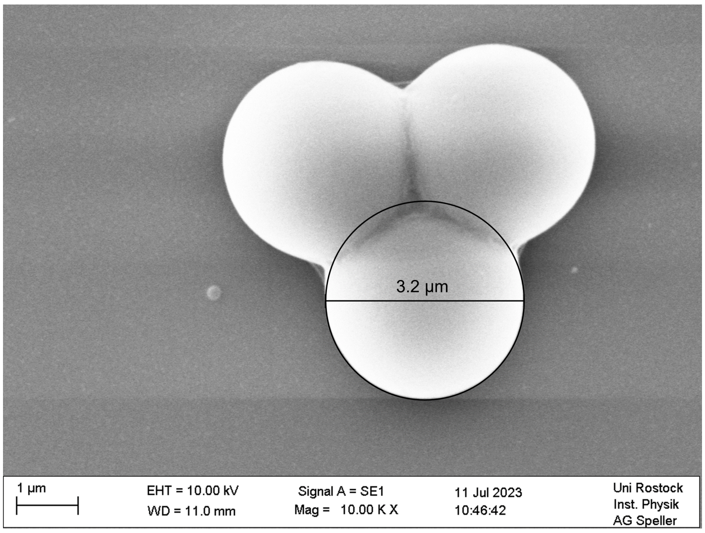

Figure S17: SEM measurement showing the 3 µm microspheres with acetone vaporisation.

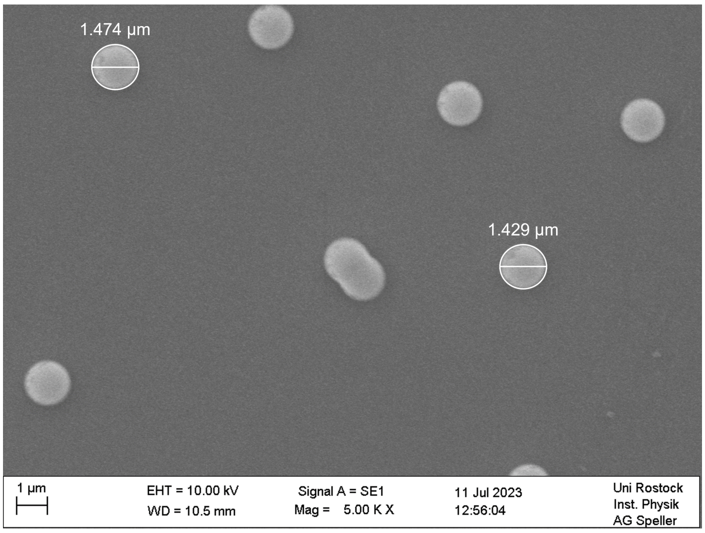

Figure S18: SEM measurement showing the 1 µm microspheres with acetone vaporisation.

The 1 µm microspheres exhibited a more pronounced effect from acetone vaporisation (Figure S18). Their diameters increased significantly, with values exceeding 1.4 µm, representing an approximately 40% increase over the target value. In the central region of Figure S18, two microspheres are visibly fused together, further illustrating the modifications induced by vaporisation. The projected areas of these spheres now approximate ellipses with numerical eccentricities of $\varepsilon = 0.27$ (top) and $\varepsilon = 0.32$ (bottom).

S7 Details of the imaging dishes and SICM setup

An AFM measurement and the corresponding line scan of the '35 mm imaging dish with a polymer coverslip bottom and low walls' (Cat. No.: 80136, IBIDI) are shown in Figure S19. The dotted line on the left indicates the location from which the line profile was extracted.

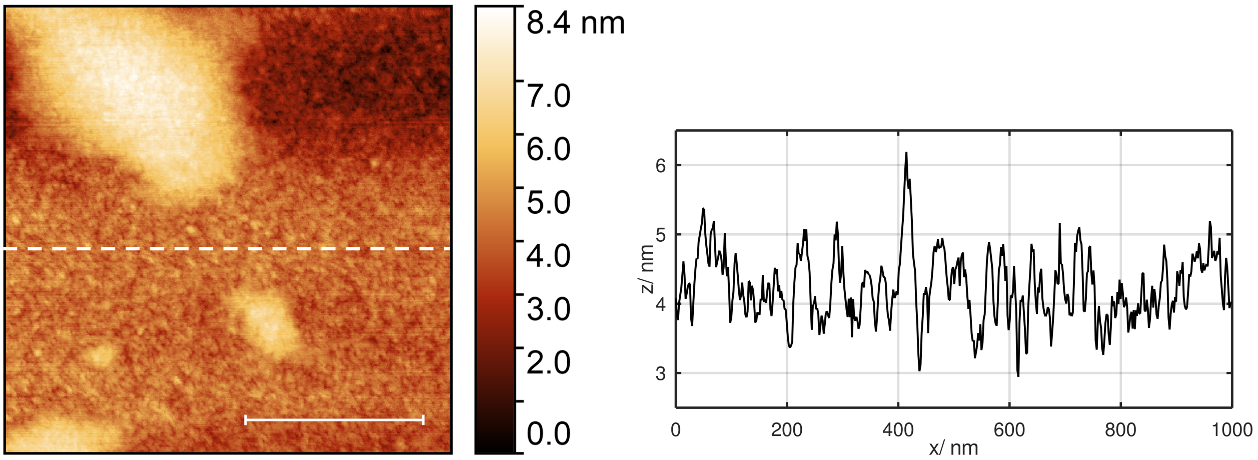

Figure S19: AFM topography (left) and exemplary line profile of a dust and water free area (right) of the bioinert cover slip of the '35 mm imaging dish with a polymer coverslip bottom and low walls' by non-contact AFM in ambient air conditions, 512 · 512 pixels, t = 30 min; the higher artefacts are adsorbed water and dust. Scale bar: 400 nm.

The working principle of the SICM is schematically illustrated in Figure S20, which also shows an SEM measurement of a nanopipette tip. The sample is placed in a Petri or imaging dish. The nanopipette is controlled by piezo actuators (control system), while the sample is positioned using the xy-table. It is viewed using the inverted optical or the fluorescence microscope.

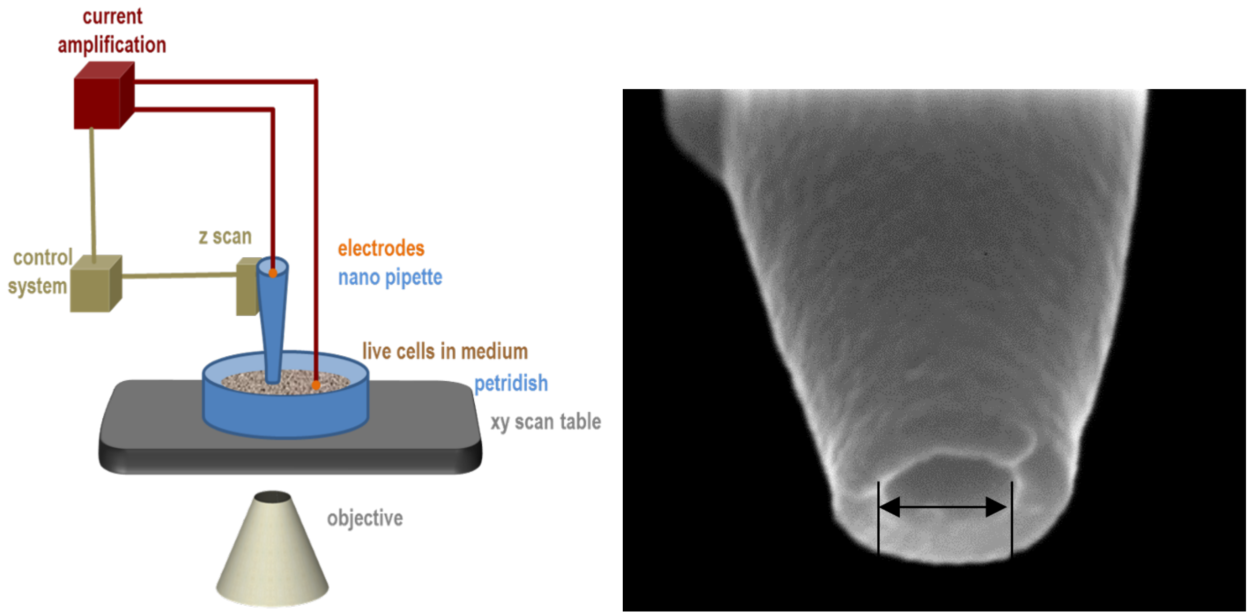

Figure S20: Schematic representation of our SICM setup (left) and SEM measurement of the tip of a nanopipette (right). Scale bar: 45 nm. Figure S20 was adapted from the report published as: Speller, S.; David, R. *Traditio et Innovatio* – Special Issue of the Magazine of the University of Rostock, 2018, pp. 12–13. https://www.uni-rostock.de/storages/unirostock/UniHome/Presse/Magazine/Traditio_Sonder_2018_screen_final.pdf. This content is not subject to CC BY 4.0.

S8 SEM measurements and other details of the nanopipettes

The pulling process and resulting geometric parameters of the nanopipettes used in this study are summarised in Table S1 and Table S2. The geometric properties of the pipettes were characterised using SEM (Figure S21 and Figure S22).

Since the pipette puller does not allow for the direct control of the opening diameter and tapper length, other parameters are adjusted instead [Sutter Instrument Company, P-2000 Laser-Based Micropipette Puller System Operation Manual - Rev. 2.4D (20201217). https://www.sutter.com/manuals/P-2000_OpMan.pdf (accessed February 25, 2025)]. The pipette puller is not calibrated. Therefore, unitless numbers are used to compare different protocols. Two distinct pulling programs were used, resulting in nanopipettes with opening diameters of approximately 45 and 100 nm. The nanopipettes required for SICM measurements were fabricated using a laser-based pipette puller P-2000 (Sutter Instrument Company, USA). In this process, a CO_2 laser heats the midpoint of a glass capillary, while two opposing masses exert force on the ends of the capillary, shaping it into a fine-tip pipette.

Table S1: Parameters for the nanopipettes used for mitochondria isolated by mechanochemical protocol.

Heat	260
Filament	4
Delay	225
Velocity	50
Pull	140
taper length	approx. 5.5 mm
opening diameter	approx. 45 nm

Table S2: Parameters for the nanopipettes used for mitochondria isolated by nitrogen cavitation.

Heat	260
Filament	4
Delay	255
Velocity	50
Pull	100
taper length	approx. 4 mm
opening diameter	approx. 100 nm

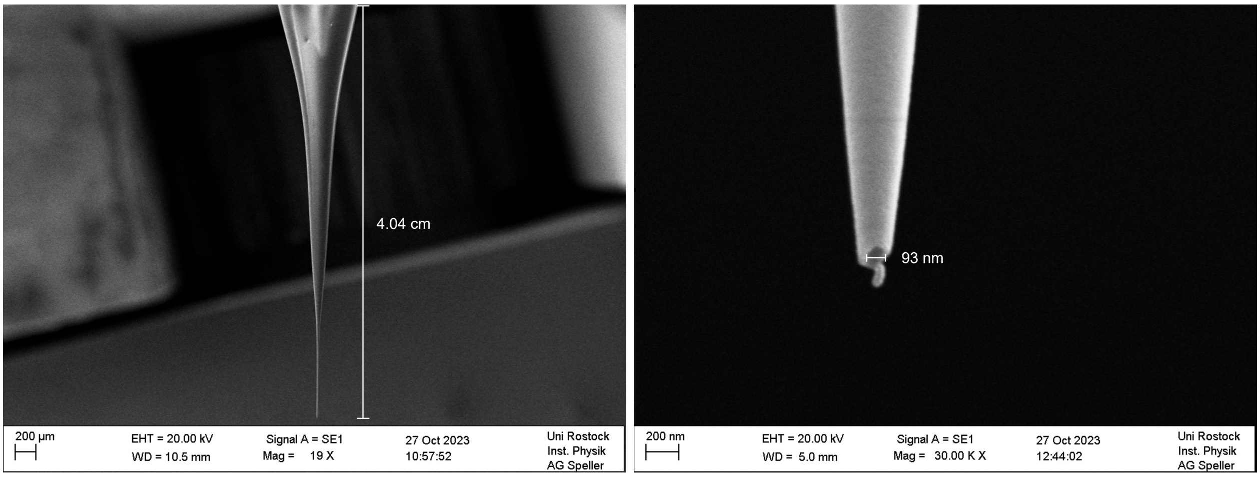

Figure S21: SEM measurement showing a nanopipette for the nitrogen cavitation protocol. It tapers within 4.04 cm and has an opening diameter of 93 nm.

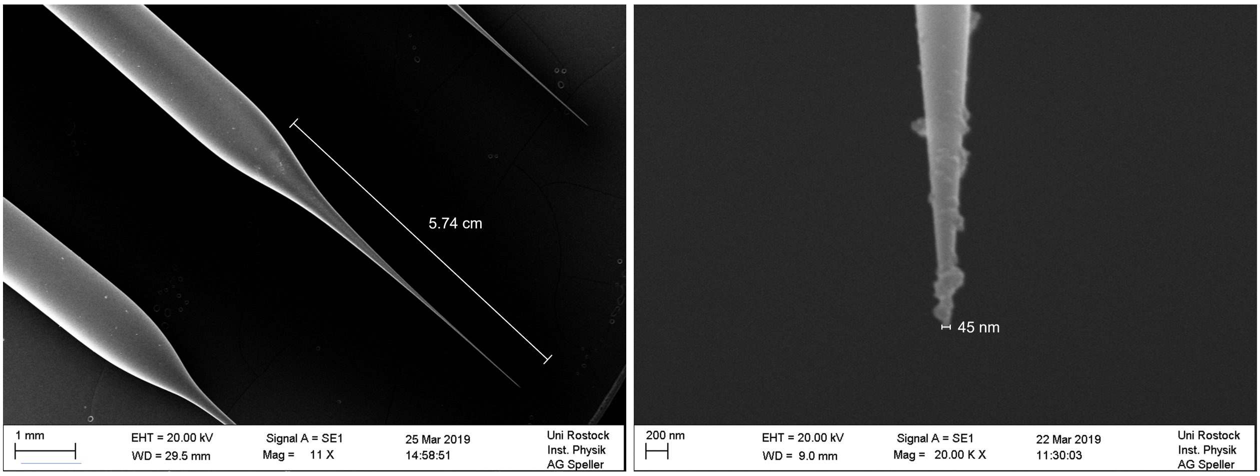

Figure S22: SEM measurement showing a nanopipette for the mechanochemical protocol. It tapers within 5.74 cm and has an opening diameter of 45 nm.

S9 Further information about the hopping mode at our SICM setup

We utilised a commercial SICM system (NX-Bio, Park Systems, Korea) for our measurements. The parameters for the hopping mode are summarised in Table S3. This mode is primarily defined by the hopping frequency, which depends on the number of pixels per image edge and the hopping height. The threshold parameter must be carefully selected: A value that is too small increases the susceptibility to electrical noise, while a value that is too large risks a closer approach, thereby increasing the probability of a pipette crash. We aimed to minimise measurement time by reducing the hopping height and control height whenever possible. The retract, approach, and fine approach steps were optimised for speed to achieve the shortest feasible measurement time. However, increasing the measurement speed excessively introduces hydrodynamic effects and pipette vibrations, which compromise measurement quality. Due to the time-critical nature of the measurements, we did not use signal averaging to avoid significantly prolonging the measurement time. Default settings were maintained for parameters such as approach delay, xy-move delay, current min, and current max.

A bias voltage of approximately 0.15–0.40 V was applied, yielding a standby current of approximately 1 nA during each measurement.

The hopping frequency f_H, which defines the measurement speed (number of pixels measured per second), is calculated as the ratio of the total number of pixels n to the measurement time t. For instance, in the case of Figure 3a, the measurement covered an area of 2.1 µm · 2.1 µm with a resolution of 128 · 128 pixels ($n = 16,384$). Simultaneously, two measurements are performed. Given a hopping height of 2 µm, the measurement required approximately 30 min ($t = 1,800$ s).

$$
\begin{aligned}
f_H &= \frac{2 \cdot n}{t} \\
&\approx \frac{2 \cdot 16,384}{1,800 \text{ s}} \\
&\approx 18.2 \text{ Hz}
\end{aligned}
$$

The exact metadata were extracted using the software Gwyddion, which also provides the feature scan

rate s. This rate represents the time per scan line. To calculate the hopping frequency, s is multiplied by the number of pixels per line (n_L) multiplied with 2, as we performed two measurements.

$$
\begin{aligned}
f_H &= s \cdot n_L \cdot 2 \\
&= 0.074 \text{ Hz} \cdot 128 \cdot 2 \\
&= 18.944 \text{ Hz}
\end{aligned}
$$

Table S3: Parameters of the hopping mode for the measurements of the mitochondria and microspheres.

parameter	value or range	description
threshold	2%	Specifies the relative current drop by which the setpoint must decrease for the termination of the approach.
control height	0.7 µm - 2.0 µm, 3.5 µm (for 3 µm microspheres)	Specifies the value by which the pipette retracts after the current threshold has been reached (also called hopping height).
quality	custom	Various presets for the following parameters can be chosen. Custom enables user specific input.
retract step	0.005 µm ms^{-1}	Specifies the speed at which the pipette should retract to the control height at the end of the approach process.
approach step	0.003 µm ms^{-1}	Specifies the speed at which the pipette should approach to the sample until half of the threshold is reached.
averages	1 time	Specifies how often a pixels is measured (and therefore how many values are to be averaged) before a value is saved and the next pixels is measured.
approach fine step	0.001 µm ms^{-1}	Specifies the speed at which the pipette should approach to the sample after half of the threshold has been reached.
approach delay	100 µs	Time delay before next approach.
xy move delay	100 µs	Time delay before xy movement.
current min	100 pA	Defines the minimum current that enables a measuring process.
current max	5,000 pA	Defines the maximum current that enables a measuring process.
